# Non-clinical Pharmacology of YTX-7739: a Clinical Stage Stearoyl-CoA Desaturase Inhibitor Being Developed for Parkinson’s Disease

**DOI:** 10.1007/s12035-021-02695-1

**Published:** 2022-01-20

**Authors:** Daniel F. Tardiff, Matthew Lucas, Iwona Wrona, Belle Chang, Chee Yeun Chung, Bertrand Le Bourdonnec, Kenneth J. Rhodes, Robert H. Scannevin

**Affiliations:** 1 Yumanity Therapeutics, 40 Guest Street, Suite 4410, Boston, MA 02135 USA; 2 Black Diamond Therapeutics, 1 Main Street, Cambridge, MA 02142 USA; 3 iNeuro Therapeutics, 325 Vassar Street, Cambridge, MA 02139 USA; 4 Deciphera Pharmaceuticals, 200 Smith St, Waltham, MA 02451 USA; 5 Pfizer Rare Disease Research Unit, 1 Portland Street, Cambridge, MA 02139 USA; 6 Verge Genomics, 2 Tower Pl, San Francisco, CA 94080 USA

**Keywords:** Stearoyl-CoA desaturase, Parkinson’s disease, α-Synuclein, YTX-7739, Pharmacology, Fatty acid desaturation

## Abstract

Stearoyl-CoA desaturase (SCD) is a potential therapeutic target for Parkinson’s and related neurodegenerative diseases. SCD inhibition ameliorates neuronal toxicity caused by aberrant α-synuclein, a lipid-binding protein implicated in Parkinson’s disease. Its inhibition depletes monounsaturated fatty acids, which may modulate α-synuclein conformations and membrane interactions. Herein, we characterize the pharmacokinetic and pharmacodynamic properties of YTX-7739, a clinical-stage SCD inhibitor. Administration of YTX-7739 to rats and monkeys for 15 days caused a dose-dependent increase in YTX-7739 concentrations that were well-tolerated and associated with concentration-dependent reductions in the fatty acid desaturation index (FADI), the ratio of monounsaturated to saturated fatty acids. An approximate 50% maximal reduction in the carbon-16 desaturation index was observed in the brain, with comparable responses in the plasma and skin. A study with a diet supplemented in SCD products indicates that changes in brain C16 desaturation were due to local SCD inhibition, rather than to changes in systemic fatty acids that reach the brain. Assessment of pharmacodynamic response onset and reversibility kinetics indicated that approximately 7 days of dosing were required to achieve maximal responses, which persisted for at least 2 days after cessation of dosing. YTX-7739 thus achieved sufficient concentrations in the brain to inhibit SCD and produce pharmacodynamic responses that were well-tolerated in rats and monkeys. These results provide a framework for evaluating YTX-7739 pharmacology clinically as a disease-modifying therapy to treat synucleinopathies.

**Supplementary Information:**

The online version contains supplementary material available at 10.1007/s12035-021-02695-1.

## Introduction

There are currently no disease-modifying therapies for Parkinson’s disease or any major neurodegenerative disease. A lack of therapeutic targets that directly impinge on underlying cellular pathologies is one of the multiple factors contributing to the challenge of developing effective therapeutics. A successful, broadly applicable disease-modifying therapy should target a protein or process central to onset or progression of human disease. In Parkinson’s disease, the small lipid-binding protein, α-synuclein, (encoded by SNCA) holds such a central position where deposits of α-synuclein in Lewy Bodies are a ubiquitous pathological hallmark of disease [1, 2]. Despite the rare occurrence of naturally occurring SNCA mutations in the general population [3], the contribution of α-synuclein to Lewy bodies in both genetically defined and idiopathic Parkinson’s disease, as well as related synucleinopathies such as dementia with Lewy Bodies (DLB) and Multiple Systems Atrophy (MSA), and the extensive validation in in vitro and in vivo model systems, places α-synuclein at the center of these human neurodegenerative diseases. Based on this central role, therapeutic approaches are being explored to pharmacologically modulate α-synuclein itself or its functional interactions with key biological pathways thought relevant to disease mechanism [4]. Despite significant effort, the critical need for new therapies that mitigate α-synuclein-dependent neuronal toxicity has yet to be realized.

Stearoyl-CoA desaturase (SCD) was identified as a potential target for synucleinopathies through unbiased phenotypic screening and target deconvolution in yeast models [5]. SCD introduces a double bond into 16- and 18-carbon fatty acyl-CoA molecules to generate monounsaturated fatty acyl-CoA chains that are incorporated into diverse lipid species, such as phospholipids, triacylglycerides, or cholesterol esters [6]. Reducing levels of unsaturated fatty acids alters membrane properties by increasing lipid packing and reducing fluidity. Unbiased lipidomic analyses of cells or mice expressing toxic α-synuclein showed that accumulation of lipid droplets, unsaturated fatty acids, and triglycerides is a conserved pathological feature of α-synuclein models from yeast through mice [7]. The ability of SCD inhibition to mitigate α-synuclein toxicity phenotypes in multiple independent reports and disease models speaks to the robustness of the protective benefit of reducing monounsaturated fatty acids [5, 7–10]. Recent electron microscopic evaluation of Lewy body pathology in Parkinson’s disease has identified early-stage Lewy bodies to consist of membranous organelles with aggregated α-synuclein [11]. These so-called pale bodies are believed to be the precursor form of fibrillar Lewy bodies suggesting a potential catalytic role for membrane lipid fatty acid composition in the α-synuclein proteinopathy cascade [12]. Identifying a drug target in the fatty acid biosynthetic pathway is thus consistent with the extensive evidence supporting a physiological role for α-synuclein in lipid biology and vesicle trafficking, which when interfered with can also lead to pathological states [12–14].

Recent in vivo validation of SCD as a target for Parkinson’s disease was obtained in a mouse model of α-synuclein toxicity. This model is based upon on the familial E46K α-synuclein mutation where two additional lysine mutations create a “3K” α-synuclein variant [15, 16]. This enhanced version of a clinically relevant form of α-synuclein has increased affinity for acidic phospholipids that results in disrupted dynamic equilibrium between membrane-bound monomers and physiological tetramers [15]. In the 3K α-synuclein mouse model, SCD inhibition was neuroprotective and restored the tetramer to monomer ratio, reduced pathological, phosphorylated α-synuclein, and ameliorated motor deficits [16]. Taken together, multiple recent reports support the hypothesis that inhibiting SCD normalizes α-synuclein and membrane interactions to restore vesicle dynamics and ultimately improves neuronal survival and motor deficits.

While SCD is a known therapeutic target that has been explored for a diverse set of lipid-based or metabolic disorders, such as diabetes, obesity, NASH, and cancer [6, 17], the above-described studies were the first to link SCD as a therapeutic target for synucleinopathies. Based on our unbiased discovery of SCD inhibitors in yeast and their protection against α-synuclein toxicity in neurons [5], we established a drug discovery program to identify novel potent, brain-penetrant SCD inhibitors for the treatment of Parkinson’s disease. Through this effort, we identified YTX-7739 as a lead clinical candidate with favorable drug-like properties. YTX-7739 has completed initial preclinical toxicology studies and is being evaluated in phase 1 clinical trials (https://www.trialregister.nl/trial/8258, https://www.trialregister.nl/trial/9172). An essential aspect of the discovery program for YTX-7739 was to characterize pharmacokinetic (PK) and pharmacodynamic (PD) properties in multiple preclinical laboratory species. Here, we describe a comprehensive set of pharmacology studies that define the relationship between SCD inhibition by YTX-7739 and modulation of fatty acid profiles in Sprague Dawley rats and cynomolgus monkeys that informed clinical program design.

## Methods

### Compounds

YTX-7739 was synthesized using standard synthetic methods and purified to 95–98% purity. Compound identity was confirmed using standard spectroscopic methods such as mass spectrometry and nuclear magnetic resonance. CAY10566 was purchased from Cayman Chemical (Ann Arbor, Michigan, product no. 10012562). Compounds were stored as powders at 4 °C and resuspended as 100% DMSO stocks at 10 mM, which were stored at − 20 °C.

### SCD Enzyme Assay

The SCD biochemical assay has been described previously (Vincent et al., 2018). In brief, SCD-induced rat liver microsomes (RLM) were isolated from mice fed a high-carbohydrate, low-fat diet for 5 days [18, 19]. RLM were then used in an optimized biochemical assay using C17:0-CoA as a substrate (Avanti Lipids, #870,717) and Rapid-Fire mass spectrometry to quantify conversion to C17:1-CoA. The RLM assay was performed at PureHoney Technologies (Billerica, MA).

### M17D Cell Line Treatment

An M17D neuroblastoma cell line that was engineered to express the 3K α-synuclein variant was used to assess effects of YTX-7739 on C16 and C18 desaturation index. The 3K α-synuclein transgene is under control of a doxycycline-inducible promoter. For these studies, doxycycline was not added so as to simply monitor the effects of YTX-7739 on fatty acids. M17D cells were grown in EMEM/F12 (1:1) media supplemented with 10% tetracycline-free FBS with 5% CO_2_ and seeded at 300,000 cells per well in 6-well PDL-coated plates and allowed to grow overnight. The next day, YTX-7739 was prepared as fivefold concentrates and added to the culture media resulting in dilution of the compound to the target concentrations. DMSO alone was added as a control at a consistent concentration of 0.1%. After 72 h of compound treatment, media was removed, the cells were washed once with PBS, cells scraped off the plate in 80% methanol, and then placed at − 80 °C until fatty acid profiling.

### Single-Dose Pharmacokinetic Studies

#### Rat Pharmacokinetics

Rat pharmacokinetic studies were performed by ChemPartner (Shanghai, China) in an IACUC-accredited facility with studies approved by their ethics committee. YTX-7739 was administered at 1 mg/mL intravenously (IV) in the foot dorsal vein (formulated in 10% DMSO/10% Solutol HS 15/80% HP-β-CD in saline). YTX-7739 was administered orally (PO) by oral gavage at 1, 3, and 10 mg/mL (formulated in 0.5% methylcellulose in water) to achieve 10, 30, and 100 mg/kg. Approximately 150 µL blood/time point was collected at each timepoint into K_2_EDTA tube via the tail vein. Blood samples were placed on ice and centrifuged to obtain plasma sample (2000 g, 5 min under 4℃) within 15 min. For terminal brain assessment, at designed timepoints post dose, animals were anesthetized and brains harvested stored at − 70℃ until analysis. Samples were then prepared for YTX-7739 analysis by protein precipitation and then analyzed using standard LC–MS/MS methods (API6500, Qtriple) and compared to a reference standard. Brain levels of YTX-7739 were determined by homogenizing tissue and protein precipitation prior to LC–MS/MS analysis. YTX-7739 concentrations were then compared to a standard curve of YTX-7739 spiked into naïve brain homogenate. Pharmacokinetic (PK) parameters were then determined using WinNonlin.

#### Cynomolgus Monkey Pharmacokinetics

Pharmacokinetic studies in non-naïve cynomolgus monkeys (minimum 7-day washout between studies) were performed by WuXi Apptec in an IACUC accredited facility and approved by their ethics committee. YTX-7739 was administered IV at 1 mg/mL and formulated in 10% DMSO/50% PEG400/40% (20% HP-β-CD in saline). Oral doses were formulated in 0.5% CMC-Na, 0.2% Tween 80 in water as a homogenous opaque suspension at 2 and 6 mg/mL. YTX-7739 was administered either through IV or orally and blood (via peripheral vessel) or CSF (cerebrospinal fluid; via a catheter implanted in the cisterna magna of conscious cynomolgus monkeys without any sedation) was withdrawn at indicated time points. Animals were observed after dosing and periodically throughout duration of study. The blood was collected into K_2_EDTA tubes and immediately processed for plasma with centrifugation (3000 × *g* for 10 min at 4 °C). CSF was taken at 0.1 mL/time point. Samples were stored at − 60 °C until analysis. The plasma was protein precipitated with an internal standard and analyzed by LC–MS/MS-05-SMBA (API 4000). For CSF, precipitated with an internal standard before LC–MS/MS analysis. YTX-7739 was then quantified relatively to a standard curve and PK parameters determined using WinNonlin software.

### Rat Pharmacology Studies

All rat pharmacology studies were performed by ChemPartner (Shanghai, China), and experimental parameters are summarized in Table 1. In all rat studies, vehicle (0.5% MC in water) or YTX-7739 formulated in 0.5% MC in water was administered at the appropriate concentrations to achieve the indicated doses with a 10 mL/kg dose volume. In each study, rats were administered YTX-7739 for the indicated days of dosing and sacrificed 4 h after the final dose. The exception is the PD duration study, where animals were sacrificed at the indicated timepoints precisely. Throughout the studies, animals were observed daily for any clinical symptoms of SCD inhibition or poor tolerability and had free access to food and water. The plasma and tissue were recovered as described above for the pharmacokinetic studies. Samples were then stored at − 80 °C. The skin was removed from the abdomen after perfusion. The plasma, brain, and skin were processed for bioanalysis. YTX-7739 concentrations were determined as described above for PK studies, and samples were analyzed for fatty acid profiles at OmegaQuant as described (see Fatty Acid Profiling methods).

**Table 1:** Pharmacology study details

*Species*	*Sex (M/F) (N/group)*	*Fig*	*Dose(s) (mg/kg),*^*1,2*^	*Time point(s)*	*Tissues / Biofluids (YTX-7739 & FA profiling)*^*3*^	*Other information*
Rat	M (8)	3, 4	10, 30, 60, 90	15d	Plasma, brain, skin	
Rat	M (6)	5	10, 30	14d on custom food, then 15d on YTX-7739 & custom food	Plasma, brain, skin	Standard, high fat, macadamia nut oil
Rat	M (8)	S2	0.3, 1, 3, 10	31d	Plasma, brain, skin	
Cynomolgus monkey	M (4)	6	1, 3, 10	15d	Cortex (YTX-7739), cortex, cerebellum, striatum, hippocampus, corpus callosum, spinal cord, CSF (FA profiling)	Non-naïve monkeys; Fulll PK curves on days 1 and 13
Rat	M (6)	7A,B	10	4 h, 1, 2, 3, 5, 7, 9, 11d	Plasma, brain	Vehicle and YTX-7739 cohorts per time point
Rat	M (6)	7C,E	10	2, 4, 8, 12, 18, 24, 48 (all hours)	Plasma, brain	Vehicle and YTX-7739 cohorts per time point
Cynomolgus monkey	M (5) / F (5)	7F,G	10	Predose (-7, -2) With YTX-7739 (1, 8, 15) Post last dose (17, 20)	Plasma, CSF	Non-terminal

^1^All studies performed with once-daily dosing by oral gavage

^2^YTX-7739 formulated as suspension in 0.5% methylcellulose (including 0.2% tween-80 for monkey studies)

^3^Animals perfused with saline prior to tissue collection

The high fat diet study was performed generally as described above with regard to YTX-7739 dosing and plasma and tissue sampling. Custom rat chow was generated by Research Diets, Inc. Standard, high fat, and macadamia nut oil food was made with 10, 45, and 45 kcal/gm of fat. Standard food contained 0.024 g soybean oil and 0.019 g lard per gram of the total food. The high fat food contained 0.029 g soybean oil and 0.21 g lard per gram of the total food. Macadamia nut oil food contained 0.029 g soybean oil and 0.21 g macadamia nut oil per gram of the total food. Final fatty acid compositions in each food preparation were confirmed (Supplemental Fig. 3).

### Cynomolgus Monkey Pharmacology Studies

The pharmacokinetics and pharmacodynamics of YTX-7739 were assessed in male cynomolgus monkeys at an IACUC-accredited WuXi Apptec facility with studies approved by their ethics committee. All studies with non-naïve (minimum 7-day washout from previous studies) are summarized in Table 1. YTX-7739 was formulated in 0.5% CMC-Na/0.2% Tween-80 in water at 0.2, 0.6, and 2.0 mg/mL and administered at 10 mL/kg to achieve 1, 3, and 10 mg/kg dose levels. Animals were weighed daily and observed for any potential clinical phenotypes. Biofluid/tissue sampling and YTX-7739 analysis was performed 4 h after the final dose on day 15 as described in the pharmacokinetics section. PK parameters were determined using WinNonlin. In a second study, male and female monkeys were administered 10 mg/kg YTX-7739 for 15 days followed by 5 days without drug (Table 1). Plasma and CSF YTX-7739 concentrations and fatty acid profiles were assessed.

### Fatty Acid Profiling

#### Fatty Acid Profiling

Fatty acid desaturation was measured at OmegaQuant (Sioux Falls, SD) by gas chromatography (GC) with flame ionization detection. Cell pellets, plasma, and CSF were processed as previously described [5]. Tissue samples were weighed into a screw-cap glass vial which contained tritricosanoin as an internal standard (tri-C23:0 TG) (NuCheck Prep, Elysian, MN). The tissue samples were homogenized and then extracted with a modified Folch extraction. A portion of the organic layer was transferred to a screw-cap glass vial and dried in a speed vac. After samples were dried BTM (methanol containing 14% boron trifluoride, toluene, methanol; 35:30:35 v/v/v) (Sigma-Aldrich, St. Louis, MO) was added. The vial was briefly vortexed and heated in a hot bath at 100˚C for 45 min. After cooling, hexane (EMD Chemicals, USA) and HPLC grade water were added, and the tubes were recapped, vortexed, and centrifuged help to separate layers. For both cells, biofluids, and tissues, an aliquot of the hexane layer was transferred to a GC vial. GC was carried out using a GC-2010 Gas Chromatograph (Shimadzu Corporation, Columbia, MD) equipped with a SP-2560, 100-m fused silica capillary column (0.25-mm internal diameter, 0.2-um film thickness; Supelco, Bellefonte, PA). Quantitation was performed as previously described [5].

### Statistical Analysis

The appropriate statistical analyses were performed dependent on the experimental setup and comparisons being made. In cases with multiple groups comparing fatty acids at different dose levels, a one-way ANOVA was performed with a post hoc Tukey test for multiple comparisons. Analyses were either performed in GraphPad Prism or using an R script (TukeyHSD) [20]_for the comprehensive datasets that reported *p* values for all fatty acids. In cases comparing two groups, such as time-matched vehicle and YTX-7739 groups in the onset kinetics and reversibility studies, a Student’s *t* test (two-tailed, unpaired, equal variance) was performed. These tests were performed in GraphPad Prism or Microsoft Excel. In all cases, significance was noted as *p* < 0.05 (*), *p* < 0.01 (**), *p* < 0.001 (***), and *p* < 0.0001 (****). Drug concentration versus pharmacodynamic response curves were fit using one-phase decay nonlinear regression curve in GraphPad Prism.

## Results

### In Vitro* Characterization of the SCD Inhibitor, YTX-7739*

**Fig. 1:**
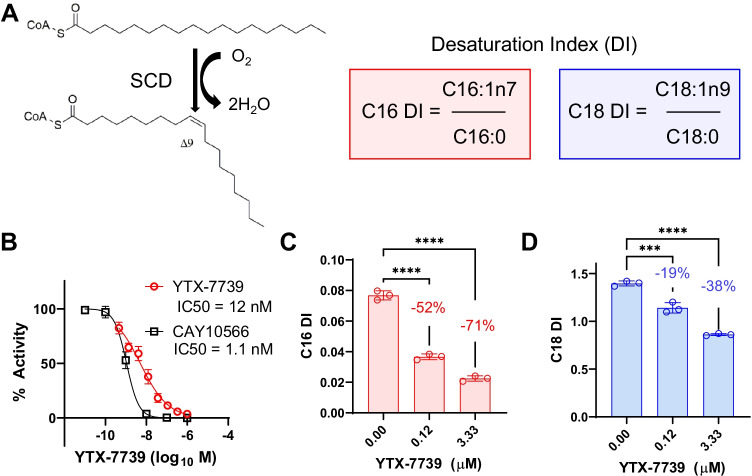
Characterization of in vitro properties of YTX-7739. **A** SCD reaction converting CoA-linked saturated to monounsaturated fatty acids of 16 (palmitoleic acid) or 18 (oleic acid) carbons in length. The desaturation index (DI) is the ratio of unsaturated to saturated fatty acids of a particular carbon chain length. **B** YTX-7739 and a reference SCD inhibitor (CAY10566) was tested in a biochemical assay using SCD-induced RLM and C17:0-CoA with Rapid-Fire mass spectrometry as an analytical readout. Data is expressed in “% activity” (*y*-axis) with concentrations in log_10_ *M* (*x*-axis). A logistic regression curve was fit to triplicate data points. The calculated IC_50_ values are inset. **C** C16 desaturation index (unsaturated C16:1n7/saturated C16:0 ratio) for M17 neuroblastoma cells treated with indicated concentrations of YTX-7739 (μM) for 3 days. Individual data points (*n* = 3) are shown with standard deviation. One-way ANOVA with a Tukey post hoc test for multiple comparisons was performed (***p < 0.001, ****p < 0.0001). Mean percent reduction is indicated. **D** C18 desaturation index (unsaturated C18:1n9/saturated C18:0) from the same M17 cell treatment. Data representation and statistical analysis is the same as in **C**

An iterative medicinal chemistry campaign identified YTX-7739 as a SCD inhibitor that reduced conversion of saturated to unsaturated fatty acids (Fig. 1A). The potency of YTX-7739 in inhibiting SCD activity was initially assessed in rat liver microsomes (RLMs) isolated from rats fed a high fat diet to induce SCD expression [18]. RLMs were then used in conjunction with a synthetic C17:0-CoA SCD substrate and rapid-fire mass spectrometry to assess enzyme activity. Using optimized reaction conditions [5], YTX-7739 inhibited rat SCD activity with an IC_50_ of 12 nM. Potency was compared to a positive reference compound, CAY10566, which inhibited SCD with a 1.1 nM IC_50_ (Fig. 1B) [21].

The activity of YTX-7739 against human SCD was then assessed in a cell-based assay. Human M17 neuroblastoma cells were administered YTX-7739 and the desaturation index (DI; unsaturated to saturated fatty acid ratio) quantified for fatty acids of 16 or 18 carbons in length (C16:0, palmitic acid; C16:1n7, palmitoleic acid; C18:0, stearic acid, C18:1n9, oleic acid). Two concentrations of YTX-7739 (0.12 and 3.33 μM) were applied to cells for 3 days. Lipids were then extracted, hydrolyzed to individual fatty acyl chains, derivatized as fatty acid methyl esters, and quantified by gas chromatography with flame ionization detection (GC-FID, OmegaQuant, Sioux Falls, SD). YTX-7739 maximally reduced the C16 DI by 71% (Fig. 1C) and the C18 DI by 38.4% at 3.33 μM (Fig. 1D). These data confirmed that YTX-7739 activity in the RLM biochemistry assay translated to SCD inhibition in a cellular environment.

### Pharmacokinetic Analysis of YTX-7739

**Fig. 2:**
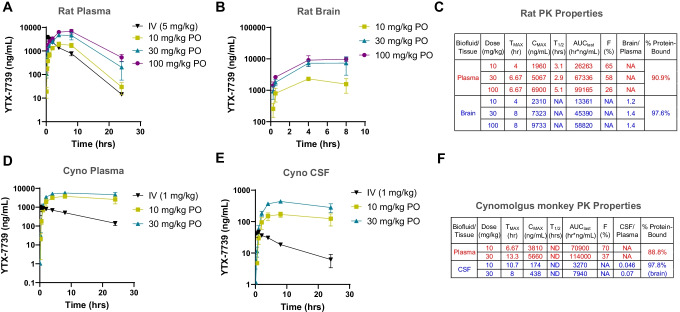
Pharmacokinetics of YTX-7739 in rat and cynomolgus monkey. **A** PK study in rats with single intravenous (IV) or oral (PO) dose of YTX-7739 at dose levels indicated in the legend. *Y*-axis is YTX-7739 concentration (ng/mL) in plasma and *x*-axis is time (hours). Each group included three rats whose blood were serially sampled. All data are mean values with error bars indicating standard deviation. **B** Rat brain PK assessment (3 rats/group) with indicated PO doses only. Each time point is a separate cohort. **C** Summary PK parameter table for rat study. Values for *T*_MAX_ (h), *C*_MAX_ (ng/mL), *T*_1/2_ (h), AUC_last_ (h*ng/mL), bioavailability (F%), brain/plasma ratio, and % protein-bound are averages of all animals in the dose group. NA indicates value was not applicable, and ND indicates value could not be determined. **D**, **E** PK study in cynomolgus monkey with single IV (1 mg/kg) or PO (10 or 30 mg/kg) doses. **D** The plasma was isolated at indicated timepoints (*x*-axis, days) and YTX-7739 levels analyzed (ng/mL, *y*-axis). Each group included three animals. **E** CSF PK of the same animals analyzed in **D**. Samples were withdrawn from an intrathecal catheter and analyzed as in **D**. **F** Summary PK parameter table for cynomolgus monkey study

Following determination of biochemical and cellular activity (Fig. 1) and drug metabolism/pharmacokinetic (DMPK) properties in vitro (data not shown), the in vivo PK of YTX-7739 was assessed in male Sprague Dawley rats and cynomolgus monkeys to determine whether in vivo drug metabolism profiles supported advancement to pharmacodynamic (PD) evaluation. In rats, a single 5 mg/kg intravenous (IV) dose of YTX-7739 was evaluated in parallel with single oral (PO) 10, 30, and 100 mg/kg doses. Plasma samples were isolated at time points through 24 h post-dosing. Multiple cohorts were administered YTX-7739 and plasma assessed throughout 24 h (Fig. 2A) and terminal compound concentrations assessed in the brain at 15 min, 30 min, 4 h, and 8 h (Fig. 2B). Whole-animal saline perfusion was performed after the blood draw and prior to tissue collection to minimize the potential contamination of YTX-7739 located within blood vessels on YTX-7739 concentrations in tissue.

YTX-7739 in rat plasma exhibited a dose-dependent and proportional 2.6-fold increase between the 10 and 30 mg/kg doses, achieving maximal concentrations (C_MAX_) of 1960 and 5067 ng/mL, respectively (Fig. 2A, C). The rat plasma protein binding of YTX-7739 was determined to be 90.9%, which correlated with a maximal achieved free concentration of 1.3 μM at 30 mg/kg (Fig. 2C). There was not a dose-proportional increase between 30 and 100 mg/kg doses as evidenced by an only 1.3-fold increase in C_MAX_ (5067 and 6900 ng/mL, respectively). The time to maximal YTX-7739 concentration (T_MAX_) occurred between 4 and 6.67 h, and the half-lives (T_1/2_) were 3 to 5 h for each dose level (Fig. 2C). In the brain, YTX-7739 achieved concentrations similar to that of the plasma with C_MAX_ values of 2310 and 7323 ng/mL for 10 and 30 mg/kg doses, respectively (Fig. 2B, C). Based on the free fraction measured from rat brain homogenate binding (Fig. 2C), YTX-7739 achieved free concentrations of 0.5 μM in the brain or approximately fivefold higher than the estimated 50% maximal response for YTX-7739 in M17 neuroblastoma cells (Fig. 1C, D). Brain exposures of YTX-7739 showed a sub-proportional increase between the 30 and 100 mg/kg doses. The T_MAX_ in the brain was slightly delayed compared to the plasma with the highest concentration achieved at 8 h. Based on total determined brain and plasma YTX-7739 concentrations, the brain-to-plasma ratio ranged from 1.4 to 1.7 for each dose level. YTX-7739 administered orally exhibited 65% bioavailability at 10 mg/kg; however, this decreased with increasing dose levels, such that at 100 mg/kg YTX-7739 showed 25.8% bioavailability (Fig. 2C).

Pharmacokinetics of YTX-7739 in cynomolgus monkeys were evaluated after single IV dose (1 mg/kg) and two oral doses of 10 and 30 mg/kg. Compound concentrations in the plasma and cerebrospinal fluid (CSF) were assessed over 24 h (Fig. 2D, E) and PK parameters determined (Fig. 2F).

YTX-7739 achieved similar concentrations in cynomolgus monkey plasma as compared to rat at 10 and 30 mg/kg (C_MAX_ = 3810 and 5660 ng/mL) dose levels. Compared to rats, there was a later T_MAX_ of 7 to 13 h (Fig. 2D,F). A reliable T_1/2_ could not be determined, as YTX-7739 levels did not decrease > 50% over the assessed 24 h (Fig. 2D). YTX-7739 bioavailability was 70% at 10 mg/kg, and this decreased to 37% at 30 mg/kg YTX-7739 (Fig. 2D, F). CSF was analyzed as a surrogate matrix for the ability of YTX-7739 to access the central nervous system (CNS). While lower compound concentrations were achieved in CSF as compared to the plasma (174 and 438 ng/mL for 10 and 30 mg/kg, respectively), the data supports YTX-7739 penetration into the CNS in non-human primates (Fig. 2E, F).

The in vivo PK studies of YTX-7739 in rats and cynomolgus monkeys indicate the compound is bioavailable, achieves concentrations predicted to engage the target SCD, has low clearance, and successfully crosses into the CNS (Fig. 2A–F). There was, however, evidence for sub-proportional exposures with higher oral dose levels of YTX-7739. Based on the biochemical potency and PK properties, YTX-7739 was advanced to evaluate PK and PD relationships in rats.

### Pharmacology of YTX-7739 in Rats

A rat study was designed to assess tolerability, PK, and PD of YTX-7739 with a repeated once-daily dosing paradigm. Multiple studies have shown that administration of SCD inhibitors cause on-target tolerability phenotypes that emerge after repeated dosing [22, 23]. Furthermore, target engagement has often been assessed through administration of a radiolabeled stearoyl-CoA and then quantifying conversion to the unsaturated oleoyl-CoA over a short time course [22, 23]. While target engagement in the liver and plasma has also been confirmed with endogenous C16 DI, the global impact of SCD inhibition has not been reported to the best of our knowledge [24]. To gain a more comprehensive overview of the effects of SCD inhibition on the desaturation index and fatty acid profiles in general, we quantified a full panel of 24 fatty acids in the plasma, brain, and skin from animals receiving YTX-7739. Compound or vehicle was administered orally, once-daily for 15 days at 10, 30, 60, and 90 mg/kg (*n* = 8/group). The blood was drawn after the final dose and plasma isolated to assess compound concentrations and fatty acid profiles. The brain and skin were isolated after whole-animal perfusion to ensure accurate quantitation of YTX-7739 and fatty acids free from contamination by the blood. While there were no reports of the eye, skin, or fur loss phenotypes, there were minor reductions in weight gain for the 30, 60, and 90 mg/kg dose groups (Supplemental Fig. 1). Despite reports of SCD inhibitor toxicity in vitro [10], YTX-7739 was well-tolerated within the tested dose range when administered for 15 days.

**Fig. 3:**
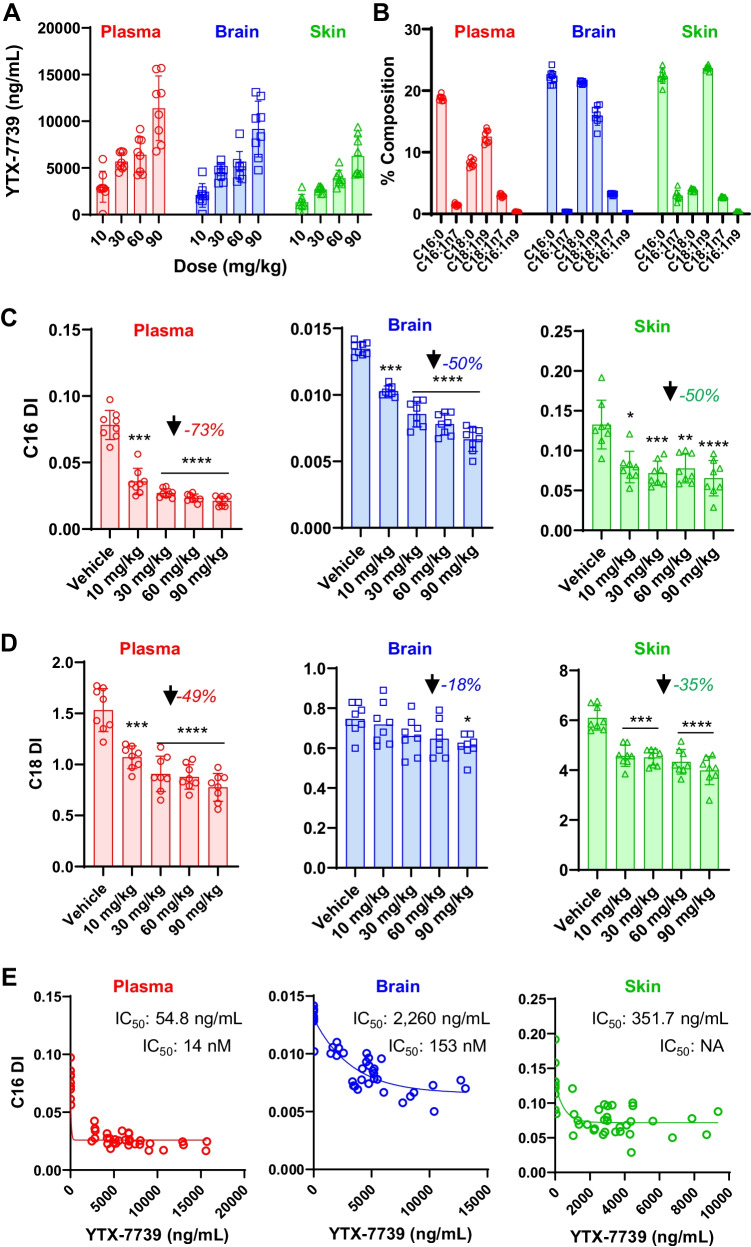
Effects of YTX-7739 on fatty acid desaturation in rats. **A** YTX-7739 concentrations (*y*-axis, ng/mL) in the plasma (red circles), brain (blue squares), and skin (green triangles). Individual data points are shown with columns indicating average values and error bars standard deviation. Dose levels are indicated below each column in “mg/kg”. **B** Percent composition (*y*-axis) of SCD substrates (C16:0 and C18:0) and products (main products: C16:1n7 and C18:1n9; metabolites: C18:1n7 and C16:1n9) in the plasma, brain, and skin for the vehicle control dose cohort only. **C** C16 DI and **D** C18 DI (*y*-axes) for the plasma (left panels), brain (middle panels), and skin (right panels). Individual data points are shown with columns indicating average values and error bars standard deviation. One-way ANOVA with a Tukey post hoc test for multiple comparisons was performed with significance noted as follows: *p* < 0.05*; *p* < 0.01**; *p* < 0.001***; *p* < 0.0001****. Inset values indicate the maximal reduction in DI for any dose as compared to vehicle. **E** Individual animal data plotted for all cohorts with associated C16 DI (*y*-axis) and YTX-7739 concentration (*x*-axis, ng/mL) from the plasma (left panel), brain (middle panel), and skin (right panel). A one-phase decay curve was fit to each full data set to determine the IC_50_ (inset, ng/mL, and free concentration in nM)

Terminal YTX-7739 concentrations were determined in the plasma, brain, and skin for each dose group 4 h after he last dose on day 15, which corresponds to the YTX-7739 T_MAX_ in rats (Fig. 3A). Similar YTX-7739 levels were achieved in the plasma and brain with slightly lower concentrations in skin (for 90 mg/kg dose group: 11,390 ng/mL in the plasma, 9170 ng/mL in the brain, and 6275 ng/mL in the skin). In each matrix, there was a dose-dependent, though sub-proportional, ~ twofold increase in YTX-7739 concentrations between doses, with exception for the 30 and 60 mg/kg doses where similar concentrations were achieved. These data were consistent with single-dose PK studies (Fig. 2C, F).

The major SCD substrates (C16:0/palmitic acid and C18:0/stearic acid) and products (C16:1n7/palmitoleic acid and C18:1n9/oleic acid) were first compared in vehicle groups to assess baseline values in each tissue (Fig. 3B). C18:1n7, an elongation product of C16:1n7, and C16:1n9, a β-oxidation product of C18:1n9, were also assessed. Among these fatty acids, C16:0 and C18:1n9 were consistently the most abundant SCD metabolites in all three matrices, while C16:1n7 was consistently the lowest (Fig. 3B). The C18:0 component of total fatty acids varied the most among matrices, where levels in the plasma (8.2%) and brain (21.3%) were higher than skin (3.9%). Based on the lower level of C18:0, the skin had a higher C18 DI (6.1) as compared to the plasma (1.5) and brain (0.75). C18:1n7 concentrations were higher than C16:1n7 in both plasma and brain, yet C16:1n9 levels were the lowest of SCD-proximal fatty acids.

C16 and C18 DI were first compared between vehicle and YTX-7739 dose groups in the plasma, brain, and skin. C16 DI was maximally reduced by 73% in the plasma and 50% in both brain and skin (Fig. 3C). The reduction in C16 DI was dose-dependent and significant at all dose levels. The changes in C16 DI were due to reductions in monounsaturated fatty acids more so than increases in saturated fatty acids. The C18 DI exhibited an overall lower magnitude of decrease with a maximal reduction of 49% in the plasma, 18% in the brain, and 35% in the skin (Fig. 3D). Whereas reduction of C16 DI in the brain was significant at all doses, only the 90 mg/kg dose group exhibited a statistically significant reduction in the brain C18 DI. As mentioned previously, C16:1n7 levels were lower than C18:1n9 (Fig. 3B). This lower abundance C16:1n7 species was also more responsive than the higher abundance C18:1n9. The differential responsiveness of C16:1n7 and C18:1n9 was consistent with results obtained in cell lines (Fig. 1C, D).

The relationship between YTX-7739 concentrations and C16 DI was explored to determine compound potency (Fig. 3E). A one-phase decay curve was fit to the terminal YTX-7739 concentration and C16 DI data for individual animals to calculate IC_50_ values in each biological matrix. These analyses revealed IC_50_ values of 54.8, 2260, and 352 ng/mL for the plasma, brain, and skin, respectively (Fig. 3E). Thus, while similar concentrations were achieved in the plasma and brain, the potency in the plasma and skin were both greater than that in the brain. Of note, the PD response achieved a maximal reduction, after which higher concentrations of YTX-7739 did not result in further decreases in C16 DI.

**Fig. 4:**
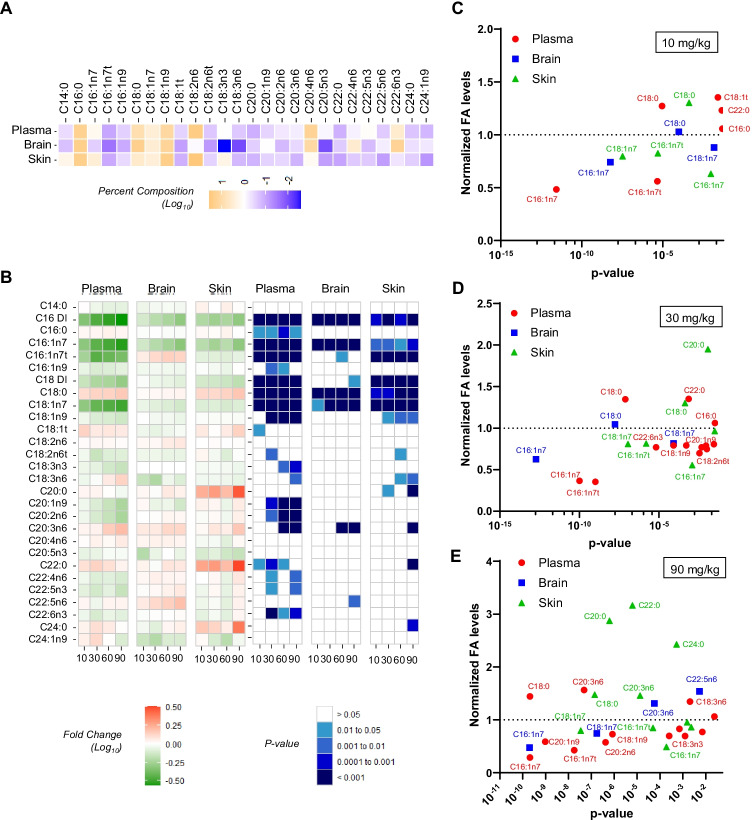
Global fatty acid profiles in rats administered YTX-7739. **A** Heat map of baseline fatty acid profiles for the plasma, brain, and skin from the vehicle cohort. Data are percent composition (log_10_) with orange indicating high and blue indicating low levels. **B** Heat maps indicating the vehicle-normalized effect size (red = increased levels; green = decreased levels) of YTX-7739 on fatty acids in the plasma (left set), brain (middle set), and skin (right set). Data are log_10_ of fold change relative to the vehicle group. Right set of panels indicate the *p* values with darker blue indicating higher significance (legend below panels). Dose levels increase from left to right (10, 30, 60, 90 mg/kg). The legends for effect size and *p* values are shown below. **C**–**E** Normalized fatty acid levels relative to vehicle (*y*-axis) and *p* values (*x*-axis) are plotted for the **C** 10 mg/kg, **D** 30 mg/kg, and **E** 90 mg/kg dose cohorts. Only fatty acids with a *p* value < 0.05 are shown on each plot with a selection highlighted in the corresponding color-coded text labels

After analyzing the response of SCD metabolites to YTX-7739, comprehensive fatty acid profiles were evaluated in the plasma, brain, and skin. The baseline profiles in the absence of compound indicated that essential fatty acids such as linoleic acid (C18:2n6) were higher in the periphery and lower in brain (Fig. 4A). Conversely, docosahexaenoic acid (DHA, C22:6n3) was highly abundant in the brain, yet low in the skin and plasma.

The responses to YTX-7739 were then characterized and both fold change (left panels, Fig. 4B) and *p* values (right panels, Fig. 4B) reported. In addition to SCD products, there were some YTX-7739 dose-dependent changes in other saturated and polyunsaturated fatty acids, especially in the plasma and skin. In the plasma, there were significant increases in C20:3n6 and decreases in C20:1n9, C20:2n6, C18:3n3, and C22:6n3. In the brain, the only consistent changes aside from SCD substrates/products were an increase in C20:3n6 at 60 and 90 mg/kg and an increase in C22:5n6 at 90 mg/kg. The skin exhibited elevations in the long chain saturated fatty acids C20:0, C22:0, and C24:0, which are extension products of C16:0 and C18:0.

To better compare the sensitivity and specificity of the responsive fatty acids, normalized fatty acid levels were compared to *p* values for each matrix in the 10, 30, and 90 mg/kg cohorts (Fig. 4C, E, only fatty acids with *p* < 0.05 are shown). SCD substrates/products, specifically C16:1n7, exhibited the most robust changes in the fatty acid profiles of each matrix and were also the most sensitive to YTX-7739. As mentioned above (Fig. 4B), additional dose-dependent changes outside of SCD substrates/products were evident and visualized when plotting normalized response and *p* values where increasing YTX-7739 dose levels resulted in additional significant changes in fatty acid levels (Fig. 4C–E). These secondary fatty acid responses were especially prominent in the plasma and skin, while secondary changes in brain were nominal.

Taken together, this study revealed that repeated dosing of YTX-7739 for 15 days was well tolerated and resulted in specific modulation of proximal SCD substrates/products at low YTX-7739 concentrations, while secondary effects on fatty acids outside of canonical SCD substrates/products were observed at higher YTX-7739 concentrations. To explore longer-term dosing paradigms and assess activity at lower dose levels, a 31-day rat PK/PD study was conducted with doses ranging from 0.3 to 10 mg/kg (Supplemental Fig. 2). The top dose of 10 mg/kg was selected based on the modest reduction in weight gain observed at 30 mg/kg in the above-described study. YTX-7739 was well-tolerated at all doses and after 31 days, there was a significant reduction in the C16 DI down to 3 mg/kg with an IC_50_ in the brain of 1052 ng/mL (Supplemental Fig. 2C-E). Thus, sustained SCD inhibition for up to 31 days with YTX-7739 was well-tolerated at concentrations associated with a robust pharmacodynamic response.

### Effects of Exogenous SCD Products on YTX-7739 Pharmacodynamic Response in Rats

Rat pharmacology studies confirmed that inhibition of SCD with orally administered YTX-7739 reduced C16 and C18 DI in brain, skin, and plasma. While these results may be reflective of SCD modulation directly within a given tissue, it is also possible that changes in circulating fatty acids. Fatty acids in the blood are obtained directly from dietary intake, or liberated from liver and adipose tissues, which could impact fatty acid levels in other tissues where fatty acids are absorbed. It is also not fully understood to what extent peripheral fatty acid biosynthesis and metabolism contributes to total fatty acid and lipid content in the brain. To address these dynamics, we explored the impact of elevated dietary concentrations of SCD products on both baseline fatty acid profiles and the YTX-7739 PD response.

**Fig. 5:**
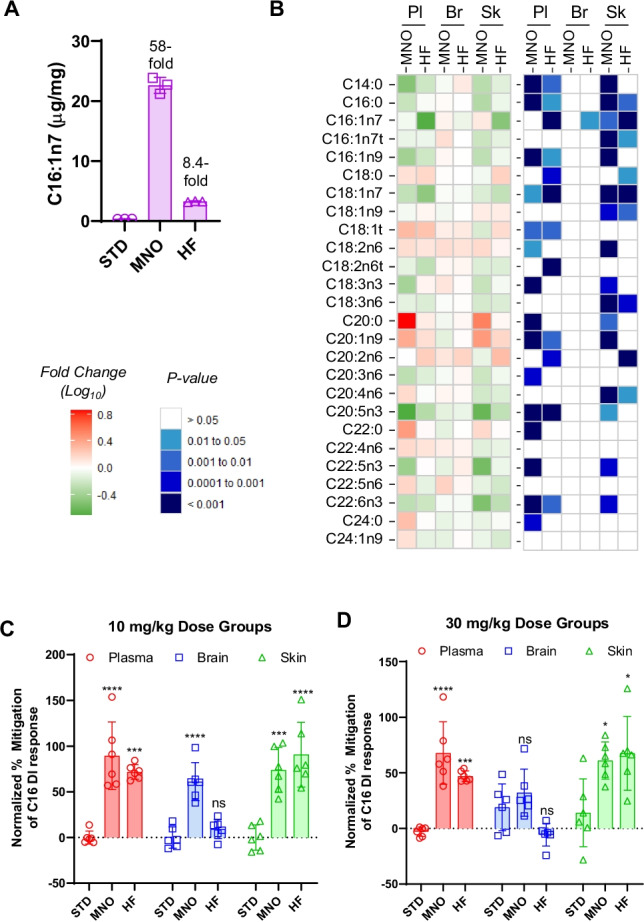
Effects of dietary C16:1n7 on C16 Desaturation Index. **A** C16:1n7 levels (μg/mg) in the three different foods (STD, standard; MNO, macadamia nut oil; HF, high fat). Fold increase relative to standard diet are shown above MNO and HF values. The mean of triplicate measurements is shown, and error bars indicate standard deviation. **B** Heat map of fatty acid profile changes (red/green, left panel) and *p* values (blue, right panel) in the plasma (Pl), brain (Br), and skin (Sk) in response to custom macadamia nut oil (MNO) and general high-fat (HF) diets. Data are normalized to standard diet. The lower left legends indicate the scale for fatty acid changes (log_10_ fold change) and *p* value groupings. **C**, **D** C16 DI (*y*-axis) was normalized to the maximal response of the standard diet group in the **C** 10 mg/kg and **D** 30 mg/kg dose groups such that 100% indicates a full mitigation of the PD response and 0% represents no change to the C16 DI response relative to the standard diet condition. Values are average of 6 rats and error bars indicate standard deviation. Statistical significance is shown for a one-way ANOVA with Tukey’s post hoc test for multiple comparisons (not significant, ns; *p* < 0.05*; *p* < 0.01**; *p* < 0.001***; *p* < 0.0001****)

Custom rat food was generated that was enriched in the SCD product C16:1n7 (palmitoleic acid), along with food containing a general high fat content. Because it was not technically feasible to directly supplement food with sufficient quantities of pure synthetic C16:1n7, we instead used macadamia nut oil (MNO), which contains high concentrations of this SCD product [25]. Direct assessment of the fatty acid profiles of the food itself confirmed this to be accurate as MNO food had a 58-fold increase in the C16:1n7 concentrations relative to standard food (Fig. 5A). There were also increases in other SCD metabolites, including C18:1n7 and C18:1n9, as well as additional long chain fatty acids (Supplemental Fig. 3). High-fat (HF) food had elevated levels of multiple fatty acids, including SCD substrates/products and many essential polyunsaturated fatty acids (PUFAs) (Fig. 5A, Supplemental Fig. 3).

Rats were first acclimated to each diet for 2 weeks prior to the initiation of compound treatment. YTX-7739 was then administered once-daily at 10 and 30 mg/kg to rats fed standard, MNO-enriched, or general high-fat food for the remainder of the 15-day study. Compared to the prior study described above, rats exhibited some additional sensitivity to 30 mg/kg YTX-7739, where there was a decrease in the extent of weight gain and some gait disruption in 3 of 6 animals (Supplemental Fig. 4A,B). The MNO diet and associated elevated levels of SCD products mitigated these responses. YTX-7739 concentrations were also assessed in the plasma, brain, and skin and shown to be comparable in each tissue for each diet (Supplemental Fig. 4C-E).

The effects of the supplemented diets were then assessed in both baseline fatty acid profiles in the vehicle cohort, as well as for effects on YTX-7739-induced changes in fatty acid desaturation. Relative to the standard diet, the MNO and HF diets caused significant and broad changes across many fatty acid species in the plasma and skin (Fig. 5B). Despite the significant increase in C16:1n7 in the MNO food itself, there was not a significant change in plasma levels of this fatty acid and only a modest, significant increase in the skin. For other fatty acids, there was a range of responses. In some cases, such as C18:1n7, there was a similar magnitude and direction of change (decrease) for both diets in both plasma and skin. In other instances, there was a change in both plasma and skin for only one diet (e.g., decrease in C22:5n3 with MNO food), or there were changes for both diets in only a single matrix (e.g., decrease in C18:3n6 and C20:4n6 in the skin). Interestingly, the baseline brain fatty acid profile was impervious to changes induced by both the MNO and HF diets (Fig. 5B, p value panel). These results suggest that either dietary fatty acids may have limited access to the brain or that there are tighter regulatory mechanisms to maintain brain lipid homeostasis as compared to the plasma or skin.

The effects of MNO and HF diets on YTX-7739-dependent reductions in C16 DI were evaluated to determine if diet supplementation of SCD products could mitigate PD responses. Data was normalized to the YTX-7739 effect in the standard diet and expressed as the percent mitigation of the C16 DI response such that deviation from “0” indicates that the diet mitigated the ability of YTX-7739 to reduce the C16 DI. At 10 mg/kg YTX-7739 (Fig. 5C), the MNO mitigated the C16 DI response by 89% in the plasma, 61% in the brain, and 74% in the skin relative to the PD responses observed in animals on the standard diet. While the HF diet similarly attenuated PD responses in the plasma and skin, this diet had no impact on C16 DI in the brain.

The 30 mg/kg dose groups demonstrated a different response. While MNO and HF diets mitigated the C16 DI response in the plasma and skin by approximately 50%, there was not a significant difference between MNO or HF diets and the standard diet response in the brain (Fig. 5D). The overall effects of the diets enriched in SCD products in the plasma and skin were also decreased at the higher YTX-7739 dose level, suggesting more extensive SCD inhibition requires additional exogenous C16:1n7 to have the same effect. These data indicate that diet supplementation of SCD products could mitigate YTX-7739’s effect on C16 DI in the periphery, while the brain appears more resistant to these effects. Only MNO-containing food, which had the highest levels of C16:1n7 (Supplemental Fig. 3), exhibited a partial reduction in the brain response at 10 mg/kg YTX-7739 and no effect at 30 mg/kg. The more modestly attenuated YTX-7739 response in the brain suggests that, while fatty acids do traverse the blood–brain barrier, they likely contribute a lower percentage to baseline fatty acids as compared to peripheral tissues. It was also notable that the HF diet, which had a more modest eightfold increase of C16:1n7 in the food itself and was unable to affect the baseline C16 DI in the brain yet did impact C16 DI in the periphery. Importantly, the data support that the reduction in C16 DI observed with YTX-7739 administration is due to local SCD inhibition and not secondary effects in the brain due to systemic SCD inhibition.

### Pharmacology of YTX-7739 in Nonhuman Primates

YTX-7739 pharmacology was next explored in cynomolgus macaques to confirm translation of the PD response in a species evolutionarily closer to humans. YTX-7739 was administered to cynomolgus monkeys (*n* = 4/group) orally, once-daily for 15 days at 1, 3, and 10 mg/kg. The doses were selected based on results from both the rat PK/PD (Fig. 3) and the cynomolgus monkey PK studies (Fig. 2D–F). YTX-7739 was well-tolerated without any adverse events or abnormal clinical findings over the course of the study. Based on the long half-life (Fig. 2D, F), full plasma PK was performed on day 1 and day 13 to evaluate whether YTX-7739 accumulated with repeat dosing. The PK profiles were similar on both days with regard to C_MAX_ and half-life, yet the day 13 PK showed presence of residual YTX-7739 at the first two timepoints from the day 12 dose (Supplemental Fig. 5A). Despite the YTX-7739 remaining from the prior dose, there was no further substantial increases in C_MAX_, AUC, or evidence of accumulation with from days 1 to 13 (Supplemental Fig. 5A).

**Fig. 6:**
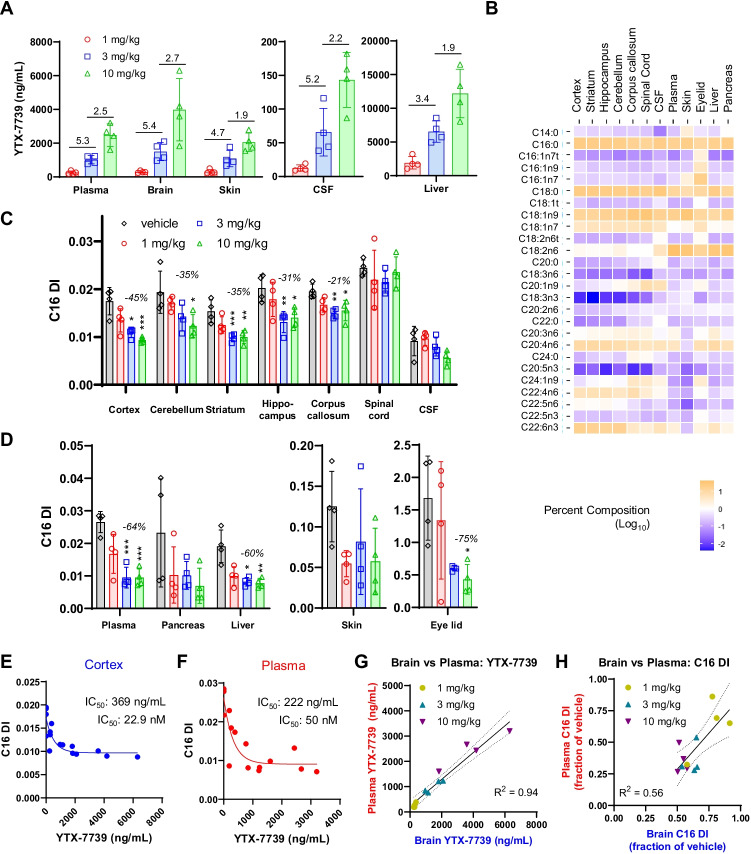
Fatty acid profiles in cynomolgus monkeys administered YTX-7739. **A** YTX-7739 concentrations (*y*-axis, ng/mL) determined in biofluid or tissue as noted below the graph. Dose levels are indicated by color and marker shape. Three different panels are shown based on different ranges of YTX-7739 concentrations. Columns are group averages with error bars indicating standard deviation. Inset values between adjacent dose levels indicate the average fold change in YTX-7739 concentrations between dose levels. **B** Heat map of baseline fatty acid profiles from different sources obtained from the vehicle cohort (below). Values (log_10_ of percent composition) are average of 4 animals. Blue indicates lower abundance fatty acids and orange indicates higher abundance fatty acids. The tissues/biofluids are indicated at the top of the heat map and individual fatty acids to the left. **C**, **D** The C16 DI (*y*-axis) for all **C** brain regions (including spinal cord and CSF) and **D** peripheral tissues in the bottom three panels separated due to C16 scales. Individual data points are shown with columns reflecting group means and error bars standard deviation. One-way ANOVA with a Tukey post hoc test for multiple comparisons was performed with significance noted as follows: *p* < 0.05*; *p* < 0.01**; *p* < 0.001***. Inset values indicate maximal % reduction in C16 DI for any dose. **E** C16 DI (*y*-axis) as a function of YTX-7739 concentration (*x*-axis, ng/mL) for the cortex. **F** C16 DI and YTX-7739 concentrations for plasma as in **E**. IC_50_ values determined by fitting a one-phase decay curve are shown in inset text in both total (ng/mL) and free (nM) concentrations. **G** The correlation of YTX-7739 concentrations (ng/mL) in the plasma versus the brain for individual animals is shown at each dose level. A linear fit and associated 95% confidence interval and *R*^2^ value are shown. **H** Correlation between the plasma and brain C16 DI response is shown for individual animals from each cohort. Data is normalized and plotted as fraction of vehicle. Trendline and *R*^2^ are shown

Four hours after the final dose on day 15, animals were sacrificed, and biofluids/tissues harvested for assessment of YTX-7739 concentration and fatty acid profiles. The 10 mg/kg YTX-7739 dose achieved comparable maximal concentrations in the plasma, brain, and skin ranging from 2050 to 3976 ng/mL, while YTX-7739 concentrations were significantly lower in CSF (143 ng/mL) and higher in the liver (12,195 ng/mL) (Fig. 6A). Dose-dependent increases in terminal YTX-7739 concentrations were observed, yet the proportionality differed across dose levels (Fig. 6A). All matrices except the liver exhibited a supra-proportional ~ fivefold increase in terminal compound concentrations between 1 and 3 mg/kg YTX-7739, while increasing the dose from 3 to 10 mg/kg afforded near dose-proportional 1.9–2.7-fold increase between dose levels in each matrix (Fig. 6A, Supplemental Fig. 5B).

Baseline fatty acid profiles from the vehicle cohort were evaluated in the brain, peripheral tissues, plasma, and CSF. While only the cortex was used for determining compound levels, additional brain regions (cerebellum, striatum, hippocampus, and corpus callosum) and spinal cord were evaluated to determine the effects of YTX-7739 on fatty acid responses (Fig. 6B). There were baseline differences in fatty acid profiles between CNS and peripheral tissues, as well as some unique features within brain regions. At baseline, all brain regions, including corpus callosum and spinal cord, had lower levels of essential polyunsaturated fatty acids C18:2n6, C18:3n3, C18:3n6, and C20:5n3 as compared to the peripheral tissues and plasma, while there were higher levels of C22:4n6. The white matter corpus callosum and spinal cord exhibited higher levels of saturated C20:0, C22:0, and C24:1n9, while lower levels of C22:6n3 and C22:5n6, as compared to other brain regions, suggestive of different composition due to the distinct structure and cellular composition of white matter. The abundance of the essential fatty acid, linoleic acid (C18:2n6), was higher in the peripheral tissues and lower in all brain regions, consistent with results in rat (Fig. 4A). Eyelid, which is an important anatomical region containing the Meibomian glands that secrete meibum, an oil responsible for lubricating the eye, exhibited higher levels of C16:1n7 than all other tissues. Excessive reduction of SCD products in the Meibomian glands has been linked to reduction of lubricating oils and excess compensatory tear production that results in eye irritation [26].

The effects of YTX-7739 on C16 DI in tissues and biofluids were then evaluated (Fig. 6C, D). In the brain, there were dose-dependent and statistically significant C16 DI reductions cortex (− 45%), cerebellum (− 35%), striatum (− 35%), hippocampus (− 31%), and corpus callosum (− 21%) (Fig. 6C). There were no significant C16 DI decreases in the spinal cord or CSF. In the periphery, the plasma (− 64%) and liver (− 60%) exhibited statistically significant and dose-dependent reductions in C16 DI, whereas in the pancreas and skin there were numerical decreases in C16 DI, yet they were not statistically significant due to high variability among individual animals (Fig. 6D, Supplemental Fig. 5C). Eyelid, which had a baseline C16 DI ten-fold higher than in the skin, had a significant reduction only at 10 mg/kg dose (− 75%) despite significant intragroup variability (Fig. 6D, right panel). In all cases, the decrease in C16 DI solely was due to a decrease in C16:1n7 and not changes in C16:0. Except for a reduction in C16:1n9 in the cerebellum, there were no additional dose-dependent or statistically significant changes in any gray matter brain region for any fatty acid, including the C18 DI (Supplemental Fig. 5C). There were also reductions in C22:0, C24:0, and C24:1n9 in the corpus callosum (Supplemental Fig. 5C). In the peripheral tissues, there were reductions of C18:1n7 in the plasma and liver, increases in saturated fatty acids (C20:0, C22:0, and C24:0) in the eyelid, and increases in C20:0 and C20:1n9 in the skin (Supplemental Fig. 5C). Thus, similar to rat studies, there were some modest secondary fatty acid changes in peripheral tissues while changes in brain were restricted to SCD products such as C16:1n7.

The relationships between terminal compound concentrations and C16 DI were then evaluated to compare potency in the brain and plasma. SCD inhibition by YTX-7739 reduced C16 DI with IC_50_’s of 369 ng/mL cortex (Fig. 6E) and 223 ng/mL in the plasma (Fig. 6F). Other brain regions exhibited comparable potency (Supplemental Fig. 5). Thus, the reduction of C16 DI in cynomolgus monkey brain occurred at lower concentrations than in rat, which had an IC_50_ of 2268 ng/mL (Fig. 3E).

Understanding the relationship between the brain and plasma PD is important to inform human clinical studies and translation of the PD since measuring a PD response is not possible in the human brain with direct fatty acid profiling. The correlation between the brain and plasma YTX-7739 concentration and C16 DI were thus compared for individual animals in each dose cohort. There was a strong correlation (*R*^2^ = 0.94) between YTX-7739 concentrations in the plasma compared to that achieved in the brain, indicating YTX-7739 concentrations in the plasma accurately predict concentrations achieved in the brain for individual animals (Fig. 6G). The reduction in C16 DI was also compared for individual animals between the plasma and brain. As for YTX-7739 concentrations, there was a strong correlation (*R*^2^ = 0.56) between the C16 DI response in the plasma and brain for individual animals. These data indicate that the plasma accurately predicts YTX-7739 concentrations and 16 DI response in the brain. Thus, measurements in the plasma may serve as a surrogate matrix to predict human responses in brain.

### Onset and Reversibility Kinetics of Pharmacodynamic Response in Rats and Nonhuman Primates

Data presented thus far indicate that 15 days of once-daily YTX-7739 administration reduces C16 DI in the peripheral tissues, brain, and plasma in multiple species. The design of these studies, however, did not to capture the kinetics of pharmacodynamic changes of the C16 DI response in terms of both onset of the responses once dosing is initiated and reversibility once dosing is stopped. We therefore performed two studies to characterize these parameters in rats and cynomolgus monkeys to both help understand dynamics of fatty acid desaturation, as well as to inform dosing paradigms in future preclinical or clinical studies.

**Fig. 7:**
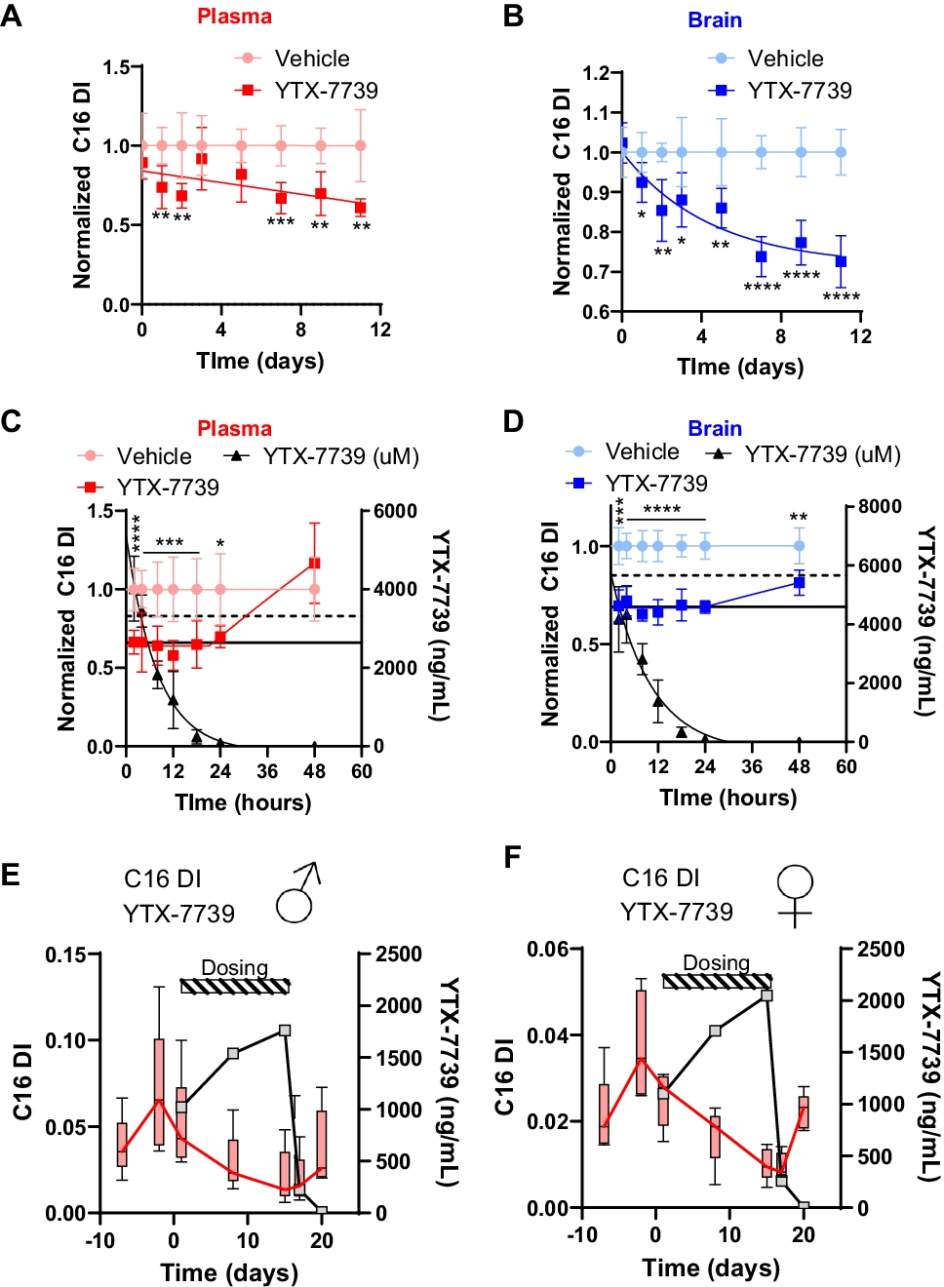
Pharmacodynamic response onset and reversibility with YTX-7739. Normalized **A** plasma and **B** brain C16 DI (*y*-axis, each animal normalized to vehicle mean) from time-matched vehicle and 10 mg/kg YTX-7739-treated cohorts of rats for indicated number of days (*x*-axis). Each data point is average of 6 animals, and error bars indicate standard deviation. Statistical significance was determined with a Student’s *t* test comparing time-matched vehicle and YTX-7739 cohorts (*p* < 0.05*; *p* < 0.01**; *p* < 0.001***; *p* < 0.0001****). Normalized **C** plasma and **D** brain C16 DI (*y*-axis, normalized as in **A** and **B** from time-matched vehicle and 10 mg/kg YTX-7739-treated cohorts of rats. *X*-axis is time (hours) after the final dose on day 11 of treatment. Secondary *y*-axis is YTX-7739 concentration (ng/mL), which is indicated in black. Both plasma and brain were fit with a two-phase decay curve with a plateau. YTX-7739 was fit with a one-phase decay curve. Each data point in **C** and **D** is average of 6 animals with error bars reflecting standard deviation. Horizontal lines indicating the maximal PD response (solid line) and the half-maximal change in C16 DI (dashed lines). Statistical significance determined as in **A**, **B**. Note, significance symbols are shown above the control line instead of the treatment line. Averaged (*N* = 5/group) plasma C16 DI (*y*-axis) for **E** male and **F** female cynomolgus monkeys over time (*x*-axis, days). Dosing interval is noted. Average YTX-7739 concentrations (right *y*-axis, ng/mL) are also indicated

A rat study was performed in which time-matched cohorts of vehicle and YTX-7739-treated animals (10 mg/kg) were dosed daily through 11 days. Time-matched cohorts were then sacrificed at various time points and C16 DI was evaluated in the plasma and brain 4 h after dosing on day 1, then again 4 h after dosing on days 2, 4, 5, 7, 9, and 11. In the plasma, the maximal response in terms of absolute difference from vehicle controls and statistical significance was obtained after dosing for 7 days (Fig. 7A). The brain was also assessed, and similar results obtained; 7 days were required to achieve the maximal reduction in C16 DI (Fig. 7B). These results suggest the intrinsic turnover rate of SCD products are relatively slow and endogenous pools need to be depleted before the effects of SCD inhibition can be detected.

A second rat study was conducted to characterize the persistence and reversibility of the C16 DI response after cessation of dosing. Time-matched, paired cohorts were administered vehicle or 10 mg/kg YTX-7739 for 11 days and then fatty acid profiles and YTX-7739 concentrations were analyzed at 2, 4, 8, 12, 18, 24, and 48 h for separate cohorts after the final dose. In the plasma, the reduction of C16 DI fully persisted for 24 h after the last dose, a time at which YTX-7739 concentrations had been reduced by 99%. At 12 h after the final dose, 66% of compound was cleared from plasma. By 48 h, the C16 DI had fully recovered to baseline levels (Fig. 7C). The response in the brain was distinct from that in the plasma. In the brain, the suppressed C16 DI persisted longer and only achieved a 50% recovery towards baseline at the 48-h time point as opposed to the full recovery observed in plasma (Fig. 7D). The clearance of YTX-7739 from the brain followed a similar kinetic profile as plasma where 67% of compound was eliminated 12 h after the final dose and 98% by 24 h after the final dose. These data indicate that the C16 DI response to YTX-7739 is reversible in the plasma and brain, yet the effect persists longer in the brain, and suggests that there are altered rates of replenishing the endogenous desaturated fatty acid pools. Determination of YTX-7739 concentrations indicated clearance rates were similar between plasma and brain.

The onset and durability kinetics were then assessed in a non-terminal cynomolgus monkey study in which the plasma was analyzed for YTX-7739 concentrations and fatty acid profiles. The design for this study differed from the terminal study in that, rather than having a vehicle control, pre-dose C16 DI was used for comparison in the same animals that were administered YTX-7739. Both males and females were administered 10 mg/kg YTX-7739 for 15 days and plasma sampled every 2 days, including additional time points at 2 and 5 days after the final dose. Compared to pre-dose values, all males displayed time-dependent reductions in C16 DI (Fig. 7E, Supplemental Fig. 6A) that reached a maximal effect at 15 days. The aggregated data was generally variable, however, and limited the interpretation of statistical significance (Fig. 7E). Additionally, there was one male monkey that had considerably higher baseline pre-dose C16 DI values and elevated values at all assessed time points (Supplemental Fig. 6). In females, there was a similar time-dependent reduction in C16 DI in 4 of the 5 animals (Fig. 7F, Supplemental Fig. 6B). Of note, the pre-dose day -2 timepoint values were markedly higher for all evaluated fatty acids as compared to the day 7 values, and this unexplained variability complicated statistical analyses and interpretation comparing pre- and post-dose fatty acid levels. Nonetheless, 9 of 10 animals showed a reduction in C16 DI between day 1 and 15 (Fig. 7E, F, Supplemental Fig. 6). At 2 days after the final dose, the C16 DI in both males and females remained low despite a 90% reduction in YTX-7739 concentrations (Fig. 7E, F). By 5 days after the final dose, however, the plasma C16 DI had recovered to baseline levels at which point YTX-7739 concentrations had decreased by over 99%. Thus, as in rats, the decrease in C16 DI in cynomolgus monkey plasma persists after YTX-7739 has been cleared from circulation for at least one day. Taken together, these data will help inform YTX-7739 human clinical trial design by understanding the length of time compound will have to be administered to elicit a maximal pharmacodynamic response, as well as the durability of the pharmacodynamic response, which has implications for characterizing both target engagement and safety profiles.

## Discussion

Data herein demonstrate that YTX-7739 is potent, brain-penetrant SCD inhibitor that reduces unsaturated fatty acid concentrations in rats and nonhuman primates at concentrations that are well-tolerated out to 31 days of dosing. These studies also highlight the unique fatty acid profile changes in response to SCD inhibition by YTX-7739, along with the impact of dietary fatty acids on pharmacodynamics and the onset and duration kinetics of the fatty acid desaturation response to YTX-7739. Together, these data provide a framework for understanding and predicting the pharmacology of YTX-7739 in humans. Importantly, the comparable compound exposures and associated pharmacodynamic response between the plasma and brain suggest plasma C16 DI may be a translatable biomarker for human target engagement.

The ability of drug candidates to access the CNS to inhibit SCD is essential for a neurodegenerative indication, such as Parkinson’s disease. YTX-7739, which exhibited adequate bioavailability and low clearance, achieved a favorable brain-to-plasma ratio of greater than 1 in both rats and cynomolgus monkeys (Fig. 2C, F). YTX-7739 achieved a range of exposures in the brain and plasma and these compound levels were predicted to inhibit SCD based on either the total compound concentration, or the calculated free concentration, in in vitro potency measurements. For the latter, maximal concentrations were achieved that were estimated to be 25- and fivefold higher concentrations than the determined biochemical and cell-based IC_50_ values, respectively (Fig. 1 and 2). The associated PD response was similarly robust in the brain and plasma as it was in vitro despite the added complexity of organismal fatty acid metabolism influenced by both biosynthetic pathways and dietary fatty acids.

The ability to detect a response in the plasma and brain speaks to the robustness of the response despite distinct reliance on a multiplicity of pathways contributing to overall fatty acid composition. In circulating plasma, fatty acid levels are dependent on release of fats from the adipose tissue and liver, as well as dietary intake. Lipid metabolism in the brain, however, is less well-characterized [27]. The brain is exceptionally rich in lipids, which contribute to more than half of its dry weight [28]. The brain also has a unique profile where it has low abundances of some essential fatty acids, such as linoleic and γ-linolenic acids, yet higher levels of docosahexaenoic acid (DHA) and arachidonic acid (AA) as compared to the periphery [29]. This distinction was especially evident when assessing YTX-7739 response in multiple cynomolgus monkey brain regions (Fig. 6C, D). Despite these complexities, the YTX-7739 pharmacodynamic response in the brain and plasma was robust and readably detectable. Of note, SCD substrates and products are also appended to many proteins through S-palmitoylation, which regulates membrane association of proteins involved in vesicle trafficking and functionally close to α-syn [30]. Indeed, the upregulation of palmitoylation mitigates α-syn toxicity [31] and SCD inhibition can modify palmitoylation status [32]. Changes in modification of proteins would not be captured with our methods, but is another area of biology potentially impacted by SCD inhibition.

While precise contributions of dietary or de novo synthesized fatty acids to total brain lipids are not fully understood, lipid biosynthetic and metabolic enzymes, such as SCD, are expressed in multiple CNS cell types. In mice and rats, SCD1 is expressed throughout the body, while SCD2 is preferentially expressed in the brain [33, 34]. The effects we see in both peripheral and CNS tissues indicate we are inhibiting both isoforms. The regional expression of these highly similar SCD isoforms is distinct from the situation in non-rodent mammals. In guinea pigs, dogs, non-human primates and humans, SCD1 and a slightly more distant paralog SCD5, are both expressed in brain [35]. This confirmed expression of SCD in the brain supports the premise that the robust response observed for YTX-7739 on C16 DI in rat (Figs. 3, 4, 5, and 7) and cynomolgus monkey (Figs. 6 and 7) brain tissue occurs due to local SCD inhibition. Data presented herein, as well as unpublished data, support that YTX-7739 inhibits multiple SCD isoforms from rodents through humans. In the brain, the available data do not indicate which CNS cell type is responsible for the changes in fatty acid desaturation. Although SCD1 and SCD5 are expressed in the brain, SCD1 is reported to be more highly expressed in oligodendrocytes and astrocytes, while SCD5 is higher in the neurons [36, 37]. In both mice and humans, RNA-seq studies indicate that oligodendrocytes express roughly 10–20-fold higher SCD1 mRNA than neurons, with astrocytes expressing ~ 5 to tenfold higher levels of SCD1 mRNA. Thus, while neurons can synthesize their own fatty acids, it is possible that a portion of the overall effect we observe in the total brain may be due to inhibition of SCD in other cell types. It is also possible SCD inhibition in glial cells could influence neuronal fatty acid composition if there is transfer of lipids from glia to neurons [38].

One consistent observation from both rat and cynomolgus monkey studies was that there was a clear maximal reduction in the C16 DI response reached despite further increases in YTX-7739 dose and associated plasma and brain concentrations (Figs. 3E nd 6E, F). A likely explanation for this phenomenon derives from the origin of saturated and monounsaturated fatty acids contributing to the total brain fatty acid composition. While the brain can synthesize many of its own fatty acids, including SCD products and substrates, radiolabeled palmitate can enter the brain and reach steady state within hours after IV administration [39]. Moreover, essential fatty acids are imported into the brain through both passive and active transport mechanisms, where they are further modified into brain-enriched DHA and AA [27]. Thus, it is likely that, despite sustained and high levels of SCD inhibition throughout the studies, SCD substrates and products coming from diet, or liberated from the liver or adipose tissue, can access the brain and incorporate into brain lipids to some extent. Given our results with MNO and HF food in the rat diet study, it does appear fatty acids may access the brain at lower levels or with reduced kinetics as compared to peripheral tissues. Specifically, the C16 DI response was fully reversed in the plasma and skin by the elevated palmitoleic acid found in MNO and HF food, yet the C16 DI response in the brain was only partially suppressed by MNO diet (Fig. 5C, D). The HF diet, which had only an eightfold increase in C16:1n7 compared to the 58-fold in the MNO diet, had no impact on the C16 DI response in brain (Fig. 5C, D). Moreover, the plasma and skin fatty acid profiles exhibited pervasive baseline changes in response to the different diets, while the brain fatty acid profile was completely refractory to dietary influence (Fig. 5B). Thus, it is likely a combination of de novo, brain-derived fatty acids, as well as those from diet, that contribute to brain fatty acids and determine the maximal DI change that is achievable in both brain and peripheral tissues.

In addition to the source of fatty acids, the uptake, biosynthetic, and degradative kinetics of SCD metabolites contribute to the overall steady state pharmacodynamic response. The onset and duration kinetics studies in rats and cynomolgus monkey suggest that SCD products have relatively long half-lives (Fig. 7). To the best of our knowledge, the half-lives of palmitoleic and oleic acid in the brain are not known. In studies exploring fatty acid metabolism in the brain, radiolabeled fatty acids are often administered intravenously and their decay observed over time. There are estimates that rat brain PUFAs, such as DHA and AA, have 2–8% daily replacement rates in the rat brain [39]. Similarly, mouse palmitic acid (C16:0) has been estimated to have a 6-to 8-day half-life [40]. If palmitoleic acid has a comparable turnover rate, then this would be consistent with our observation that 7 days was required to achieve a 50% reduction in C16 DI in brain (Fig. 7B). There are clearly competing factors that could influence apparent fatty acid half-lives, such as modulation of pathway activity that occurs in response to SCD inhibition and impact of dietary fatty acids. Such an estimate fails to explain why a maximum reduction of 50% in C16 DI was routinely observed. It is possible, that at maximal extent of SCD inhibition, plasma concentrations of C16:1n7 are sufficient to replenish the brain levels to achieve the observed steady-state levels.

It was also repeatedly observed that C18 DI was more resistant to SCD inhibition by YTX-7739 than was C16 DI. Even in cultured cells, the reduction of C18 DI required higher concentrations of YTX-7739 to achieve a similar response (Fig. 1D). This trend was observed in the rat plasma, brain, and skin (Fig. 3C, D). For example, while YTX-7739 reduced C16 DI by 50%, C18 was only reduced by 18%. Several factors likely contribute to this conserved difference. C16:1n7 is at least tenfold less abundant than C18:1n9 in the plasma, brain, and skin of rats (Fig. 4A) and cynomolgus monkeys (Fig. 6B), and thus potentially, a more sensitive species in which even small perturbations in enzyme kinetics are more evident. This lower C16:1n7 abundance is likely at least partially due to the reduced dietary intake of C16:1n7 as compared to C18:1n9 (Supplemental Fig. 3) [41], but also potentially related to the ability of C16:0 to be converted into C18:0 and C16:1n7 be elongated to C18:1n7, a positional isomer of oleic acid (C18:1n9). We noted that C18:1n7 also responds to SCD inhibition (Fig. 4B–E), as does C16:1n9, a β-oxidation product of oleic acid. It is also possible that the C18:1n9 half-life is simply considerably longer than C16:1n7. Based on the resistance of C18:1n9 to YTX-7739-induced changes and its greater abundance compared to C16:1n7, overall levels of total combined monounsaturated fatty acids, thus largely track with C18:1n9.

The 15-day rat study revealed secondary effects of SCD inhibition on fatty acid profiles beyond changes in SCD substrates and products. At the highest dose levels and with more sustained SCD inhibition, there were secondary fatty acid changes that were unique to each tissue (Fig. 4B). These indirect changes included immediate downstream fatty acids, such as C20:1n9 in the plasma, but also multiple significant differences in PUFAs. For example, C20:2n6 was reduced in the plasma, while C20:3n6 was increased in the plasma, brain, and skin (Fig. 4B). In fact, C20:3n6 was the only other fatty acid to change in a dose-dependent manner in the brain other than C16:1n7, C18:1n7, and C18:0. Interestingly, while C20:3n6 levels were elevated, all upstream fatty acids, including γ-linolenic acid (C18:3n6) and linoleic acid (C18:2n6), were unaltered by YTX-7739. It is known that PUFAs can influence SCD expression [42]. However, it is not clear if reduced SCD activity could influence regulation of PUFA metabolism. Alternatively, a downstream consequence of SCD inhibition on membrane-related events or bioenergetics could indirectly affect PUFA metabolism and alter fatty acid profiles. The other most obvious alterations were the increase in saturated fatty acids of 20, 22, and 24 carbon length in the skin (Fig. 4B). This response was only observed at the highest concentrations and is consistent with elongation of accumulated SCD substrates C16:0 and C18:0 as their conversion to unsaturated fatty acids was blocked. It thus appears that the tissue- or biofluid-specific metabolism determines susceptibility to changes in secondary fatty acids.

Data herein provide several key insights into the relationship between YTX-7739 exposure and SCD-mediated effects on fatty acid profiles. These results provide target exposures and kinetic parameters to inform both future preclinical animal studies and human clinical studies. Of clinical relevance, these data suggest that the plasma PD response reflects the response in the brain, providing an opportunity to explore the plasma C16 DI as a translational biomarker of SCD target engagement in humans. Indeed, preclinical safety studies supported the advancement of YTX-7739 into human Phase I safety trials (https://www.trialregister.nl/trial/8258, https://www.trialregister.nl/trial/9172). In addition to the extensive validation of SCD as a target for α-synuclein toxicity in vitro, recent mouse efficacy studies confirm that reducing monounsaturated fatty acids in vivo improves motor deficits, reduces α-synuclein biochemical pathologies, reduces phospho-serine129 and lipid droplets, while promoting survival of dopaminergic neurons [16]. Taken together, extensive in vitro and in vivo analyses support the further evaluation of YTX-7739 as an SCD inhibitor for the treatment of Parkinson’s disease and provides evidence for key relationships among α-synuclein, lipid biology, and stearoyl-CoA desaturase.

## Conflict of Interest

All authors are present or past employees of Yumanity Therapeutics and have equity in the company.

## Supplementary Information

Below is the link to the electronic supplementary material.

Supplementary file1 (PDF 341 KB)

## Data Availability

Data and material can be made available upon request of the corresponding author.

## Code Availability

Code for the statistical analysis can be made available upon request of the corresponding author.
