# Supplementary material for: Non-clinical Pharmacology of YTX-7739: a Clinical Stage Stearoyl-CoA Desaturase Inhibitor Being Developed for Parkinson’s Disease
Source: Mol Neurobiol. 2022 Jan 20;59(4):2171–89. doi: 10.1007/s12035-021-02695-1 (PMC9015998; doi:10.1007/s12035-021-02695-1)
Supplement: Supplementary file 1 — Supplementary file1 (PDF 341 KB) [file 12035_2021_2695_MOESM1_ESM.pdf]

Supplemental Information: Molecular Neurobiology

Non-Clinical Pharmacology of YTX-7739, a Clinical Stage Stearoyl-CoA Desaturase Inhibitor Being Developed for Parkinson's Disease

Daniel F. Tardiff[*], Matthew Lucas[*,†], Iwona Wrona[*,†], Belle Chang[*,‖], Chee Yeun Chung[*], Bertrand Le Bourdonnec[*,‡], Kenneth J. Rhodes[*,§], Robert H. Scannevin[*,¶]

[*], Yumanity Therapeutics, 40 Guest Street, Suite 4410, Boston, MA, 02135
[†], Current address: Black Diamond Therapeutics 1 Main Street, Cambridge, MA 02142
[‡], Current address: Deciphera Pharmaceuticals 200 Smith St. Waltham, MA 02451
[§], Current address: Wave Life Sciences 733 Concord Avenue, Cambridge, MA 02138
[¶], Current address: Verge Genomics, 2 Tower Pl, San Francisco, CA 94080
[‖], Current address: iNeuro Therapeutics 325 Vassar St, Cambridge, MA 02139

Article Type: Original research

Corresponding author: Daniel F. Tardiff, dtardiff@yumanity.com, 617-409-5304

Supplemental Fig. 1: Rat body weights from 15-day once-daily YTX-7739 pharmacology study

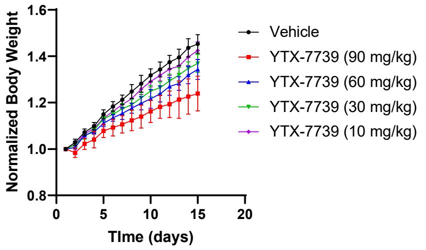

Supplemental Fig. 1 Rat body weights from 15-day once-daily YTX-7739 pharmacology study Individual rat body weights were normalized to day 1 (y-axis) and plotted as a function of time (days, x-axis). Data points are average normalized body weights and error bars indicate standard deviation.

Supplemental Fig. 2: 31-day rat YTX-7739 pharmacology study

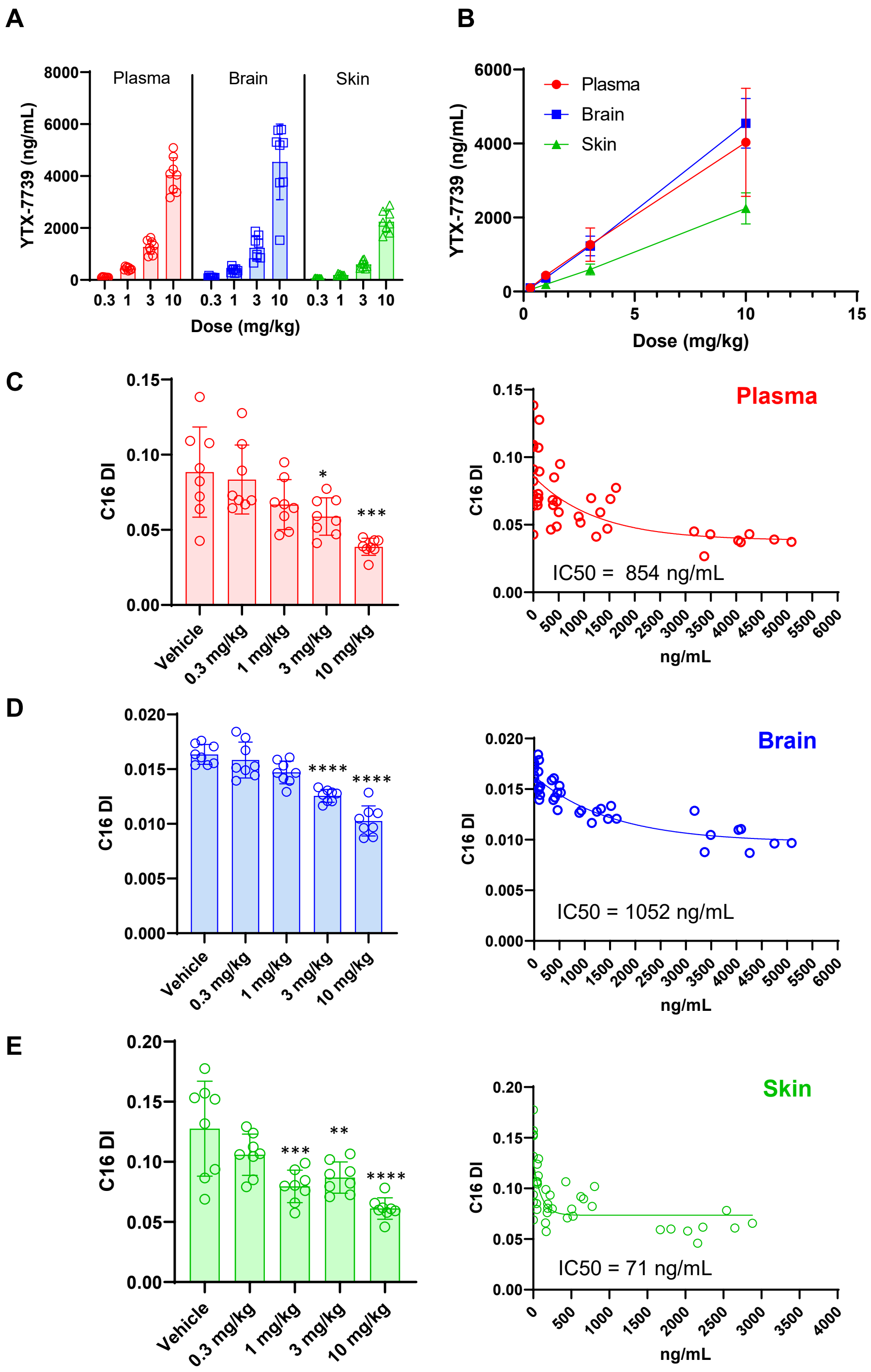

Supplemental Fig. 2 31-day rat YTX-7739 pharmacology study

(A) YTX-7739 concentrations (y-axis, ng/mL) for each dose group. Individual animal data are shown with columns reflecting group means and error bars standard deviation. Data is shown for plasma (red), brain (blue), and skin (red). (B) YTX-7739 concentrations (y-axis, ng/mL) plotted as a function of dose (x-axis, mg/kg). (C) Left panel: C16 DI (y-axis) in plasma plotted for each dose group as indicated below the columns (mg/kg). Significance was determined using a one-way ANOVA and Tukey's post-hoc test for multiple comparisons (($p<0.05$, *; $p<0.01$,**; $p<0.001$,***; $p<0.0001$,****). Right panel: C16 DI plotted as function of measured YTX-7739 concentrations (x-axis, ng/mL). A one-phase decay curve was fit and IC50 (ng/mL) determined and noted in the inset. (D) C16 DI in brain. All presentation and statistics as shown in (C). (E) C16 DI in skin. All presentation and statistics as shown in (C).

Supplemental Fig. 3: Fatty acid profile of food used in rat supplemental diet study

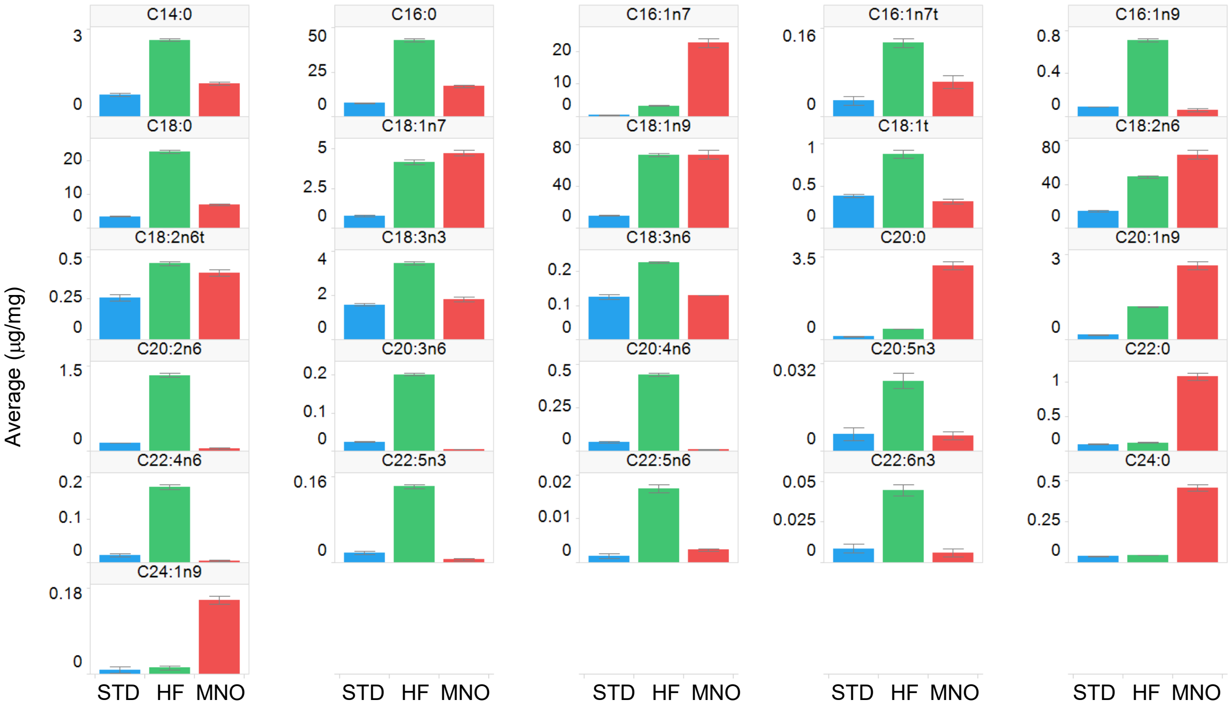

Supplemental Fig. 3 Fatty acid profile of food used in rat supplemental diet study
Samples of standard (STD), high-fat (HF), and macadamia nut oil (MNO) rat food were analyzed for baseline fatty acid compositions (y-axis, g fatty acid / mg food). Data are trellised based on fatty acid species. Columns indicate mean values of three technical replicates and error bars are standard deviation.

Supplemental Fig. 4: Supporting data for rat diet study

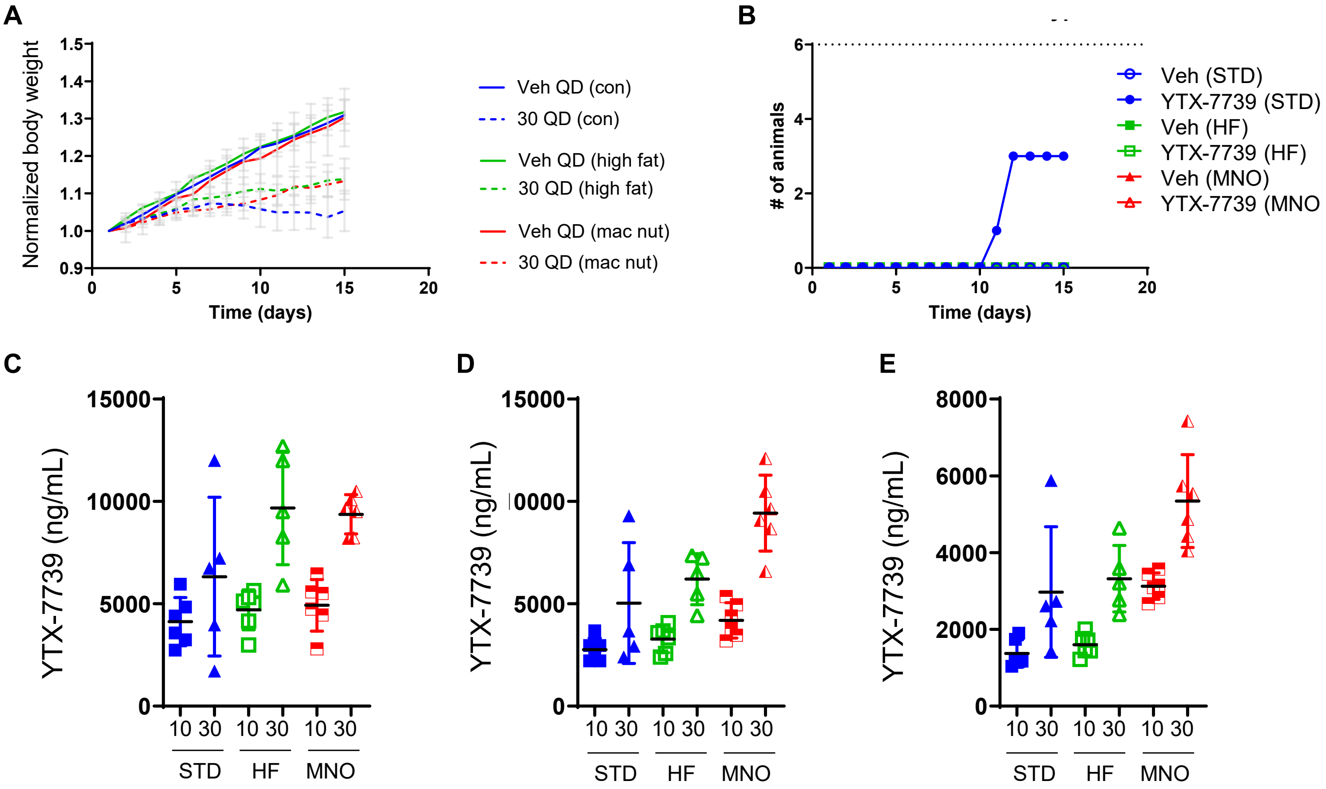

Supplemental Fig. 4 Supporting data for rat diet study

(A) Normalized body weights (y-axis) for cohorts receiving different diets (colors) and vehicle (solid lines) or 30 mg/kg YTX-7739 (dashed lines). Data are group averages (N=6 rats/group) and error bars indicate standard deviation. (B) Number of animals (y-axis) experiencing a hind limb phenotype for each cohort as depicted by the legend. Horizontal dashed line indicates the number rats per group. (C-E) YTX-7739 concentrations (y-axis, ng/mL) for each diet and dose level (10 or 30 mg/kg) for (C) plasma, (D) brain, and (E) skin. Individual animal data is shown with horizontal lines indicating group means and error bars indicate standard deviation.

Supplemental Fig. 5 Pharmacology of YTX-7739 in cynomolgus monkeys

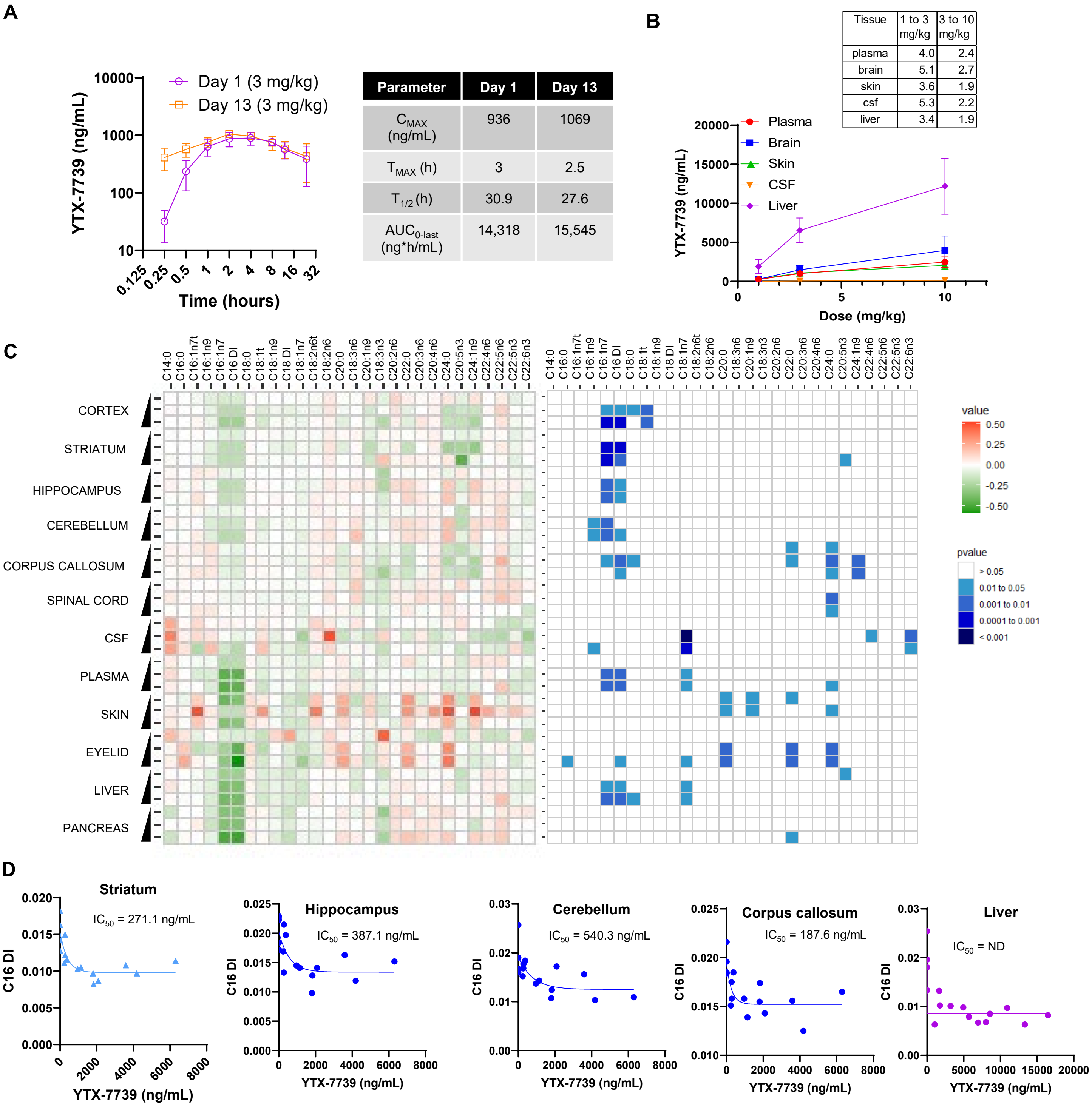

Parameter	Day 1	Day 13
C_{MAX} (ng/mL)	936	1069
T_{MAX} (h)	3	2.5
$T_{1/2}$ (h)	30.9	27.6
$AUC_{0\text{-}last}$ (ng*h/mL)	14,318	15,545

Tissue	1 to 3 mg/kg	3 to 10 mg/kg
plasma	4.0	2.4
brain	5.1	2.7
skin	3.6	1.9
csf	5.3	2.2
liver	3.4	1.9

Supplemental Fig. 5 Pharmacology of YTX-7739 in cynomolgus monkeys
(A) Day 1 and day 13 PK assessment. YTX-7739 concentrations (y-axis, ng/mL) were assessed at indicated time points (x-axis, log2 time, hrs) for 3 mg/kg YTX-7739. For day 13, the elevated concentration at 0.25 and 0.5 hrs reflects the remaining YTX-7739 from the dose administered on day 12. Data are average of 4 animals and error bars indicate standard deviation. Table contains summary data. (B) YTX-7739 concentration (y-axis, ng/mL) as a function of dose (x-axis, mg/kg) for each tissue. The fold change between adjacent dose levels is indicated in the inset table. (C) Normalized (log10) fold change for all brain regions and peripheral tissues/biofluids as compared to vehicle (left panel) and associated p-values (right panel). Tissues/biofluids and order of increasing dose are indicated on the left. Statistical significance and p-values were determined with a one-way ANOVA with a post-hoc Tukey test for multiple comparisons. (D) C16 DI (y-axis) plotted as a function of YTX-7739 concentration (x-axis, ng/mL) for additional tissues demonstrating statistically significant reductions in C16 DI (Fig. 6). Data was fit with one-phase decay curves and IC50 values determined and indicated in the inset (ng/mL).

Supplemental Fig 6: Aggregate animal data for cynomolgus monkey plasma onset and reversibility study

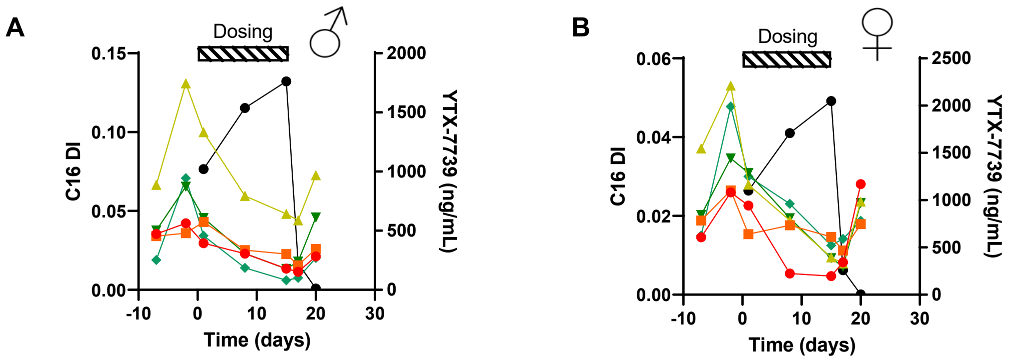

Supplemental Fig. 6 Individual animal data for cynomolgus monkey plasma onset and reversibility study

(A) Male and (B) female cynomolgus monkeys were administered YTX-7739 and assessed for compound concentration (right y-axis, ng/mL) and C16 DI (left y-axis) as a function of time (days, x-axis). Individual animal data is shown due to inherent baseline variability.
